# SSABE-TSCM: drift-aware and interpretable financial sentiment analysis for low-resource Bangla via adaptive semi-supervised and temporal contrastive modeling

**DOI:** 10.3389/frai.2026.1724407

**Published:** 2026-04-08

**Authors:** Iftakhar Ali Khandokar, Priya Deshpande

**Affiliations:** Department of Electrical and Computer Engineering, Marquette University, Milwaukee, WI, United States

**Keywords:** adaptive boosting ensemble, Bangla NLP, contrastive learning, financial sentiment analysis, low-resource language processing, semi-supervised learning, SHAP explainability, temporal drift modeling

## Abstract

Analyzing the tone of Bangla financial news is challenging because labeled data are scarce, the language is morphologically rich, and economic discourse shifts over time. We address these hurdles with a three-part framework. First, **SSABE** a *S*emi-*S*upervised *A*daptive *B*oosting *E*nsemble iteratively refines pseudo-labels, adjusts model weights by recent performance, and applies sector-aware voting to distill reliable labels from limited data. Second, the *T*emporal *S*entiment *C*ontrastive *M*odule (**TSCM**) aligns yearly embedding prototypes via contrastive loss, keeping the classifier robust against vocabulary drift and shifting economic regimes. Third, **Temporal-SHAP** yields token-level attributions that reveal how term importance changes across years and industries, thereby making the system transparent to analysts. Evaluated on a 5-year (2018–2023) Bangla financial news corpus spanning eight sectors, our pipeline attains a macro-F_1_ of 0.782 and 91.4 % explanation fidelity surpassing fine-tuned transformer and self-training baselines by 6 %–12 % absolute. Performance remains stable when labels are scarce, sectors are imbalanced, or economic shocks such as the inflation and currency decline of 2023 occur. Moreover, yearly sentiment scores and Temporal-SHAP attributions track inflation and exchange-rate trends, confirming real-world relevance. The proposed framework offers a scalable, interpretable solution for monitoring emerging-market news, supporting regulators, policymakers, and investors who rely on trustworthy Bangla-language insights.

## 1 Introduction

Understanding the “mood” of financial news is crucial because market sentiment can move prices, shape investment plans, and steer policy decisions (Pang and Lee, 2008; Cambria et al., 2017; Tetlock, 2007; Li, 2014). Most existing sentiment tools work well for English, where we have plenty of labeled data and strong pretrained models (Araci, 2019; Chen et al., 2022). In contrast, Bangla lacks large annotated corpora, comprehensive financial lexicons, and dedicated language models (Bhattacharjee et al., 2021; Hasan et al., 2021). As a result, off-the-shelf English solutions transfer poorly. This language disparity hampers the development of localized financial intelligence systems. Moreover, regional financial narratives often reflect unique macroeconomic signals and culturally specific cues. Without tailored resources, even accurate translation fails to capture sentiment shifts in context. Addressing this gap is essential for equitable access to sentiment-aware financial technologies in emerging markets. Such efforts can empower policymakers, investors, and analysts to make informed decisions grounded in localized economic narratives.

Three obstacles make Bangla financial sentiment analysis especially difficult:

1. **Few labeled examples**. Reliable annotations are hard to gather, so fully supervised training is fragile.
2. **Complex language**. Bangla has rich morphology and sector-specific jargon that confuse general NLP models.
3. **Rapid language drift**. The meaning of key terms changes with new policies, economic shocks, or sector trends, causing older models to lose accuracy (Kutuzov et al., 2018; Huang et al., 2020).

We tackle these issues with a hybrid framework that blends semi-supervised learning, time-aware representation learning, and transparent explanations:

- *S*emi-*S*upervised *A*daptive *B*oosting *E*nsemble (**SSABE**) expands limited labels with high-confidence pseudo-labels, re-weights its base models by recent accuracy, and applies sector-aware voting to stay robust in low-resource settings.
- *T*emporal *S*entiment *C*ontrastive *M*odule (**TSCM**) learns yearly “prototypes” of sentiment and keeps them distinct via contrastive loss, so the classifier remains stable even when vocabulary shifts.
- **Temporal-SHAP** gives word-level explanations that trace how the influence of key terms evolves across years and industries, offering much-needed transparency for high-stake financial decisions.

We test our system on 50,000 Bangla news articles published from 2018 to 2023 across eight economic sectors. The full model scores a macro-F_1_ of 0.782 and achieves 91.4 % explanation fidelity, beating strong neural and classical baselines. It also holds up when labels are scarce, sectors are imbalanced, or shocks such as the 2023 inflation spike change market language. These results highlight the model's adaptability to real-world challenges in low-resource financial sentiment analysis.

In short, we deliver a scalable, interpretable, and time-aware framework for Bangla financial sentiment analysis. By merging semi-supervised adaptive boosting with contrastive temporal learning and token-level explainability, our approach turns a small pool of labeled examples and a large stream of unlabeled news into reliable, up-to-date sentiment signals. The framework is designed for practical deployment in low-resource financial settings, where timely annotation and frequent retraining are infeasible. Its modular design further enables straightforward extension to other languages, domains, and evolving economic contexts.

## 2 Literature review

Bangla financial sentiment analysis faces three intertwined hurdles: limited annotated data, a linguistically complex script, and vocabulary that shifts with market events. To situate our work, we review six research strands that tackle these issues from different angles: (i) sentiment modeling for financial text, (ii) Bangla-specific NLP resources, (iii) semi-supervised learning, (iv) adaptive ensembles, (v) temporal drift modeling, and (vi) model interpretability.

Most existing studies solve only one piece of the puzzle e.g., enlarging datasets with pseudo-labels or tracking language drift leaving other challenges unmet. Very few approaches simultaneously expand training data, adapt to temporal change, and provide explanations that auditors and investors can trust.

This gap motivates our integrated framework, which couples a *Semi-Supervised Adaptive Boosting Ensemble* (SSABE) with contrastive temporal representation learning and Temporal-SHAP attribution. The combination is designed to be data-efficient, drift-aware, and transparent, making it well suited to low-resource financial domains. Section 3 details each component and how they work together.

### 2.1 Financial sentiment analysis

Financial sentiment analysis converts the language of markets into quantitative signals that guide forecasting, risk management, and investment strategy. Unlike general-purpose sentiment classification, it must decipher jargon, interpret numbers, and link text to macro–economic context.

Early rule-based work by Nasukawa and Yi (2003) introduced domain-specific sentiment extraction, while Tetlock (2007) demonstrated that the tone of financial commentary can predict stock returns, establishing sentiment as a market-moving factor. Subsequent research focused on tailored resources and models. The *Financial PhraseBank* supplies thousands of manually labeled news snippets for polarity classification (Malo et al., 2014); FinBERT adapts BERT to large financial corpora and outperforms generic models on multiple benchmarks (Araci, 2019). Architectures aimed at fine-grained reasoning such as FinQANet for question answering over earnings reports (Yang et al., 2020) and FinText for multi-task analysis of news and SEC filings (Chen et al., 2022) further refine sentiment estimates by explicitly modeling numerical volatility and event context.

Despite these advances, nearly all prior work targets English and relies on proprietary, high-volume feeds (e.g., Bloomberg, Reuters). In low-resource languages like Bangla, labeled corpora are scarce, and direct transfer of English-trained models fails to capture local expressions or sector-specific idioms. Existing systems also treat articles as independent, overlooking how sentiment evolves over time and across industries.

To close these gaps, we introduce a hybrid framework for Bangla financial news that (i) bootstraps limited annotations via semi-supervised adaptive ensembles, (ii) captures temporal dynamics through contrastive time-series modeling, and (iii) provides SHAP-based explanations for transparency. The resulting system delivers accurate, interpretable, and resource-efficient sentiment analysis suited to emerging-market contexts.

### 2.2 Bangla NLP and low-resource language challenges

Bangla is the world's seventh most widely spoken language, yet it remains a classic “low-resource” case in NLP. Large, task-specific corpora, pretrained models, and off-the-shelf pipelines that drive progress for English, Chinese, or German are still sparse for Bangla, constraining system accuracy, robustness, and domain transfer (Hasan et al., 2021).

#### 2.2.1 Early rule-based work

The first wave of Bangla NLP tackled tokenization, part-of-speech tagging, and shallow parsing with handcrafted rules or statistical models (Bhattacharya and Bandyopadhyay, 2005). These systems were brittle and difficult to extend to new domains. A summary of existing Bangla NLP resources and their limitations is presented in Table 1.

**Table 1:** Summary of existing Bangla NLP resources and their limitations.

References	Resource	Type	Domain-specific	Sentiment support	Limitations	Language scope
Sagor et al. (2020)	BNLP toolkit	Toolkit	×	×	Lacks pretrained sentiment models	Bangla
Bhattacharjee et al. (2021)	Bangla-BERT	Transformer LM	×	×	General-domain corpus only	Bangla
Kakwani et al. (2020)	IndicBERT	Multilingual LM	×	×	Not optimized for Bangla	Indic (15+) languages
Alam et al. (2020)	BanglaNews	Dataset	×	×	Small-scale; NER-focused only	Bangla
Hosain et al. (2021)	BnHateSpeech	Dataset	×	Partial	No finance domain; hate speech only	Bangla

#### 2.2.2 Transformer era resources

Recent monolingual and multilingual transformers Bangla-BERT (Bhattacharjee et al., 2021), BNLP (Sagor et al., 2020), and IndicBERT (Kakwani et al., 2020) provide stronger baselines, but they are trained mostly on general-domain text (Wikipedia, Common Crawl). Consequently, they struggle with specialized registers such as finance, healthcare, or law.

#### 2.2.3 Linguistic hurdles

Bangla's rich morphology, frequent compound formation, and parallel script styles (formal Sanskritised vs. colloquial) create tokenization and embedding inconsistencies (Islam and Rahman, 2021). The shortage of high-quality sentiment lexicons and time-aligned corpora further impedes progress.

#### 2.2.4 Limited annotated data

Available datasets e.g., Bengali Hate Speech (Hosain et al., 2021) or BanglaNews (Alam et al., 2020) cover only a handful of genres, contain modest sample sizes, and exhibit variable annotation quality. For niche domains such as financial news, labeled examples are virtually non-existent.

These gaps make conventional supervised pipelines brittle, especially when the language is figurative or the domain evolves rapidly as in finance. Our hybrid framework addresses the problem by (i) bootstrapping new labels with semi-supervised adaptive ensembles, (ii) learning time-aware representations to capture sentiment drift, and (iii) offering model explanations to build user trust.

### 2.3 Semi-supervised learning for text classification

Semi-supervised learning (SSL) exploits the natural abundance of unlabeled text to boost performance when labeled data are scarce an Achilles' heel in tasks such as Bangla financial sentiment analysis. By blending the fine-grained supervision of a small annotated set with structural cues mined from unlabeled corpora, SSL reduces overfitting and improves domain transfer (Zhu, 2005).

#### 2.3.1 Classical methods

Early SSL for NLP relied on *self-training* and *co-training*: a model iteratively labels its own unlabeled examples to enlarge the training pool (Blum and Mitchell, 1998). Although conceptually simple, these approaches suffer from confirmation bias errors compound as pseudo-labels are recycled. Graph-based techniques such as label propagation (Zhu et al., 2002) mitigate this by diffusing labels across a similarity graph, yet they struggle with class imbalance and scale poorly to modern deep encoders.

#### 2.3.2 Deep SSL era

Recent advances embed SSL inside neural architectures. *Pseudo-Labeling* (Lee, 2013) selects high-confidence predictions as additional training targets. *Consistency-regularization* variants including the Π-Model (Laine and Aila, 2017) and Mean Teacher (Tarvainen and Valpola, 2017) enforce prediction stability under data perturbations, while UDA (Xie et al., 2020) and FixMatch (Sohn et al., 2020) marry strong data augmentation with confidence filtering. These methods push decision boundaries into low-density regions of feature space, yielding state-of-the-art results on many benchmarks.

#### 2.3.3 Outstanding gaps

Most SSL frameworks apply static confidence cut-offs and treat all unlabeled instances uniformly. In financial text, however, sentiment expression varies by sector and drifts over time; fixed thresholds risk propagating noisy or outdated labels. Furthermore, models rarely track *per-sector* performance, limiting their ability to adapt to domain heterogeneity.

#### 2.3.4 Our contribution: SSABE

We introduce a *Semi-Supervised Adaptive Boosting Ensemble* (SSABE) that augments classical pseudo-labeling with three synergistic modules:

- **Dynamic confidence thresholding** adjusts the pseudo-label cut-off for each base learner according to its historical Macro-F1-score, reducing error amplification.
- **Momentum-based weighting** boosts models that sustain high accuracy over recent epochs, while down-weighting those with performance dips, yielding a more stable ensemble.
- **Sector-aware aggregation** tracks sentiment accuracy by industry sector and re-balances votes accordingly, enhancing adaptability across financial sub-domains.

Together, these mechanisms enable SSL to cope with domain shift and class imbalance, delivering cleaner pseudo-labels and stronger generalization critical for low-resource, high-stakes applications such as Bangla financial sentiment analysis.

A comparative summary of popular SSL methods and their limitations in domain-specific NLP tasks is shown in Table 2.

**Table 2:** Comparison of semi-supervised learning methods and their limitations.

References	Method	Supervision	Thresholding	Domain-aware	Augmentation	Limitations
Lee (2013)	Pseudo-labeling	Softmax confidence	Fixed	×	×	No domain variance modeling
Laine and Aila (2017)	Pi-model	Temporal consistency	Fixed	×	Noise injection	No sector-level awareness
Tarvainen and Valpola (2017)	Mean teacher	EMA targets	Fixed	×	Dropout	No adaptive weighting
Xie et al. (2020)	UDA	Consistency + TF-IDF Aug	Fixed	×	Backtranslation	Heavy augmentation cost
Sohn et al. (2020)	FixMatch	Confidence + consistency	Fixed	×	RandAugment	Ignores domain drift
**Ours**	**SSABE**	**Dynamic Voting**	**Dynamic**	✓	Moderate	Optimized for domain-shifted data

Bold values indicate the best performance among the compared methods.

As illustrated, our approach uniquely combines dynamic thresholding, momentum-based ensemble weighting, and sector-aware aggregation to overcome critical gaps in prior SSL frameworks particularly in low-resource, temporally-variant, and sentiment-sensitive domains like Bangla finance.

### 2.4 Adaptive and ensemble-based learning

Ensemble learning combines multiple models to curb variance, curb overfitting, and boost Generalization. Classical schemes bagging, boosting, and stacking exploit learner diversity to exceed the accuracy of any single component (Dietterich, 2000). In NLP, ensembles are especially useful for domains that exhibit drift, class imbalance, or noisy labels, conditions common to low-resource languages such as Bangla.

#### 2.4.1 Boosting foundations

AdaBoost adaptively re-weights training instances, forcing subsequent learners to focus on hard-to-classify examples (Freund and Schapire, 1997). Its text-specific variant, BoosTexter, demonstrated strong gains in document categorization where decision boundaries are fuzzy (Schapire and Singer, 2000). Modern deep-ensemble techniques extend these ideas to neural architectures, improving robustness to distribution shifts (Lakshminarayanan et al., 2017) and delivering calibrated uncertainty estimates for tasks like sentiment analysis and NER (DeVries and Taylor, 2018).

#### 2.4.2 Limitations

Conventional ensembles usually rely on static or uniform weights, which is suboptimal for financial news where sentiment dynamics differ by sector (e.g. banking vs. energy) and over time (e.g. pre- vs. post-crisis). Moreover, few methods handle the extra uncertainty introduced by pseudo-labels in semi-supervised settings.

#### 2.4.3 Proposed framework: SSABE

We introduce a *Semi-Supervised Adaptive Boosting Ensemble* that extends pseudo-label boosting with three novel controls:

- **Macro-F1-weighted voting** dynamically scales each model's vote by its historical Macro-F1 on a development set, providing data-driven trust calibration.
- **Momentum-based confidence penalty** decays the influence of models whose recent performance has dipped, mitigating drift.
- **Sector-aware prioritization** further tunes weights using *per-sector* validation scores, capturing domain heterogeneity in financial text.

These mechanisms allow the ensemble to adapt to temporal change, sector-specific language, and the noise inherent in semi-supervised pseudo-labels key requirements for accurate sentiment analysis in Bangla financial news.

Table 3 summarizes the characteristics of relevant ensemble learning methods in NLP, highlighting the unique aspects of our SSABE framework.

**Table 3:** Comparison of ensemble-based methods and their adaptivity and domain-awareness characteristics.

References	Method	Learning type	Adaptivity	Semi-supervised	Domain-aware	Weighting strategy
Freund and Schapire (1997)	AdaBoost	Supervised	Sample-based	×	×	Error-Focused
Schapire and Singer (2000)	BoosTexter	Supervised	Sample + Feature	×	×	Confidence scoring
Lakshminarayanan et al. (2017)	Deep ensembles	Supervised	Static	×	×	Uniform
DeVries and Taylor (2018)	Uncertainty-aware ensemble	Supervised	Attention-based	×	×	Entropy-weighted
Zhou et al. (2020)	BERT-of-Theseus	Supervised	Dynamic layers	×	×	Adaptive layer replacing
Clark et al. (2019)	Layer attention BERT	Supervised	Attention-based	×	×	Layer-wise gating
Chen et al. (2020)	Selective ensemble	Supervised	Sample filtering	×	✓	Trust-aware voting
Wang et al. (2022)	Dual-view co-training	Semi-supervised	Pseudo-labeling + filtering	✓	×	Agreement-based
Sohn et al. (2020)	FixMatch	Semi-supervised	Confidence + consistency	✓	×	Static threshold
**Ours**	**SSABE**	**Semi-supervised**	✓**(Macro-F1 + Momentum)**	✓	✓**(Sector-specific)**	**Macro-F1 and domain weighted**

Bold values indicate the best performance among the compared methods.

In summary, the proposed SSABE framework not only accommodates the label scarcity of low-resource domains but also integrates adaptivity and domain-awareness, ensuring that ensemble decisions are both context-sensitive and dynamically robust.

### 2.5 Temporal modeling in NLP

Language and the sentiment it conveys shifts over time in response to social, political, and economic events. In finance these shifts are rapid: vocabulary, tone, and contextual cues can change within days of major policy announcements or market shocks. Standard NLP models assume stationary data, so their accuracy decays when the underlying distribution drifts (Lazaridou et al., 2021).

#### 2.5.1 Timestamp-aware language models

Early work augmented n-grams or recurrent nets with explicit timestamps to capture diachronic change (Kutuzov et al., 2018). Transformer extensions such as T-BERT inject temporal tags into BERT's attention layers (Huang et al., 2020), while Temporal RoBERTa encodes calendar signals to track topic and sentiment drift in news streams (Rosin et al., 2022). Although effective, these methods rely on large, continuously time-stamped corpora and costly fine-tuning resources often unavailable in low-resource languages.

#### 2.5.2 Contrastive alternatives

Contrastive temporal learning offers a data-efficient route: a contrastive loss separates embeddings from different time windows, encouraging year- or quarter-wise clustering and improving robustness to temporal noise (Ke et al., 2022). This approach excels at detecting concept drift and mapping sentiment trajectories without massive retraining.

Our framework adopts this contrastive strategy to model Bangla financial news. By aligning representations across adjacent periods we capture evolving sentiment cues while avoiding the computational overhead of re-training large transformers on streaming data.

Building on these ideas, we propose a lightweight yet effective **Temporal Sentiment Contrastive Module (TSCM)** that captures yearly variations in financial sentiment without requiring fine-tuned transformers or continuous timestamping. The TSCM architecture comprises the following components:

- **Prototype embedding generation:** for each year *t*, we compute a mean prototype embedding μ_*t*_ from BERT representations of labeled instances:
$$\mu_{t}=\frac{1}{N_{t}}\overset{N_{t}}{\underset{i=1}{\sum }}\text{BERT}\left(x_{i}^{t}\right)$$
- **Hard negative sampling:** for each μ_*t*_, the most similar μ_*k*_ from a different year is selected as a hard negative.
- **Temporal contrastive loss:** we optimize a loss that increases the distance between prototypes from different years:
$$L_{\text{temp}}=\underset{t_{i}\neq t_{j}}{\sum }-\log\frac{\exp\left(\text{sim}\left(\mu_{t_{i}},\mu_{t_{j}^{-}}\right)\right)}{\sum_{k}\exp\left(\text{sim}\left(\mu_{t_{i}},\mu_{k}\right)\right)}$$

Table 4 compares prior temporal modeling techniques with our proposed TSCM, demonstrating its advantages in low-resource and sentiment-sensitive settings.

**Table 4:** Comparison of temporal modeling methods across contrastive, low-resource, and sentiment-aware dimensions.

References	Method	Architecture	Temporal encoding	Contrastive	Low-resource	Sentiment-aware
Huang et al. (2020)	T-BERT	BERT + temporal tags	✓	×	×	✓
Rosin et al. (2022)	Temporal RoBERTa	RoBERTa + time embedding	✓	×	×	✓
Kutuzov et al. (2018)	Diachronic embeddings	Word2Vec + time	✓	×	✓	×
Ke et al. (2022)	TimeCL	BERT + contrastive	✓	✓	×	×
Dhingra et al. (2021)	Time-conditioned BERT	BERT + time feature	✓	×	×	×
Lazaridou et al. (2021)	Temporal robustness Eval	RoBERTa + Eval bench	✓	×	×	×
Zhou et al. (2022)	TimeBERT	BERT + temporal bias adaptation	✓	×	×	×
Edmiston et al. (2022)	EventTime-BERT	BERT + temporal event heads	✓	×	×	✓
Cao et al. (2021)	Diachronic BERT	BERT (incremental)	✓	×	×	×
Wang et al. (2021)	ELMo-TD	ELMo + temporal drift module	✓	×	✓	×
Zhou et al. (2020)	BERT-of-Theseus	BERT + temporal reconfig	✓	×	✓	×
Brandl et al. (2022)	Lifelong temporal BERT	Continual BERT + time mask	✓	✓	×	×
Gonen et al. (2020)	BERT drift detection	BERT + time-wise diff	✓	×	×	×
Chalkidis et al. (2022)	LexGLUE	Legal-BERT with year signals	✓	×	✓	✓
**Ours**	**TSCM**	**BERT (frozen) + contrastive**	✓**(Year-aware)**	✓	✓	✓

Bold values indicate the best performance among the compared methods.

In summary, TSCM offers a scalable, plug-and-play module for capturing financial sentiment evolution across time. Its contrastive learning foundation, prototype-level embedding, and hard-negative alignment enable temporally aware sentiment analysis even in low-resource and partially labeled datasets.

### 2.6 Interpretability and explainability in financial NLP

In finance, model outputs influence high-value decisions subject to regulation and audit. Stakeholders therefore demand not only accurate predictions but also clear, defensible explanations (Danilevsky et al., 2020). As deep transformers become the default architecture, the need for transparent reasoning grows sharper.

#### 2.6.1 Existing explanation tools

Attention weights offer rough word-level salience maps (Bahdanau et al., 2015). Perturbation methods such as LIME approximate local decision boundaries by masking input tokens (Ribeiro et al., 2016), while gradient-based techniques like Integrated Gradients trace attribution through the network (Sundararajan et al., 2017). SHAP provides a model-agnostic alternative grounded in game-theoretic Shapley values, yielding consistent feature importance scores (Lundberg and Lee, 2017).

#### 2.6.2 Finance-specific practice and gaps

Analysts have applied these tools to highlight influential terms, uncover bias, and compare model logic with domain expertise (Bastings et al., 2019). Yet most studies treat language as static, ignoring how feature importance shifts across market cycles or industry sectors. Without a temporal lens, explanations can become stale or misleading when sentiment vocabularies evolve.

#### 2.6.3 Our contribution: temporal SHAP

We embed a *Temporal SHAP Attribution* module in our pipeline. Instead of analyzing a single model snapshot, we compute SHAP values for yearly checkpoints and track token-level contributions over time. This reveals:

- **Shift detection**: emerging keywords whose influence rises or falls with economic events.
- **Sector drift**: terms that carry different sentiment weight in banking vs. energy news.
- **Explanation stability**: periods where attribution patterns remain consistent, signaling model reliability.

By aligning interpretability with temporal modeling, we provide auditors and domain experts with explanations that evolve alongside the market, strengthening trust in our Bangla financial sentiment system.

- Shifts in sentiment-driving terms (e.g., “policy” changing from positive to negative).
- Sector-specific lexical importance (e.g., “merger” being positive in banking but neutral in energy).
- Attribution stability across years, useful for flagging model inconsistencies.

Table 5 compares major interpretability frameworks and highlights the novelty of our temporal SHAP extension.

**Table 5:** Comparison of explainability and interpretability methods across model-agnostic, token-level, and temporal-aware dimensions.

References	Method	Model-agnostic	Token-level attribution	Temporal-aware	Domain-specific	Main limitation
Bahdanau et al. (2015)	Attention weights	×	✓	×	×	Not always faithful
Ribeiro et al. (2016)	LIME	✓	✓	×	✓(customizable)	Sensitive to perturbation
Sundararajan et al. (2017)	Integrated gradients	×	✓	×	×	Requires differentiability
Lundberg and Lee (2017)	SHAP	✓	✓	×	Partial	Computationally expensive
Bastings et al. (2019)	Differentiable binarization	×	✓	×	×	Requires custom architecture
Chen et al. (2022)	FinText (financial classifier)	✓	✓	×	✓	Uses static lexicons
Poerner and Schütze (2018)	Contextual decomposition	×	✓	×	✓	Hard to scale
Jin et al. (2020)	BERTology interpretations	×	✓	×	×	Limited to BERT-family
DeYoung et al. (2020)	ERASER benchmark	✓	✓	×	Partial	Lacks temporal evaluation
**Ours**	**Temporal SHAP**	✓	✓	✓	✓	Requires temporal partitioning

Bold values indicate the best performance among the compared methods.

In summary, the integration of temporal SHAP not only enhances model transparency but also supports longitudinal error analysis and regulatory justification in sentiment-sensitive financial environments.

## 3 Methodology

**Figure 1:**
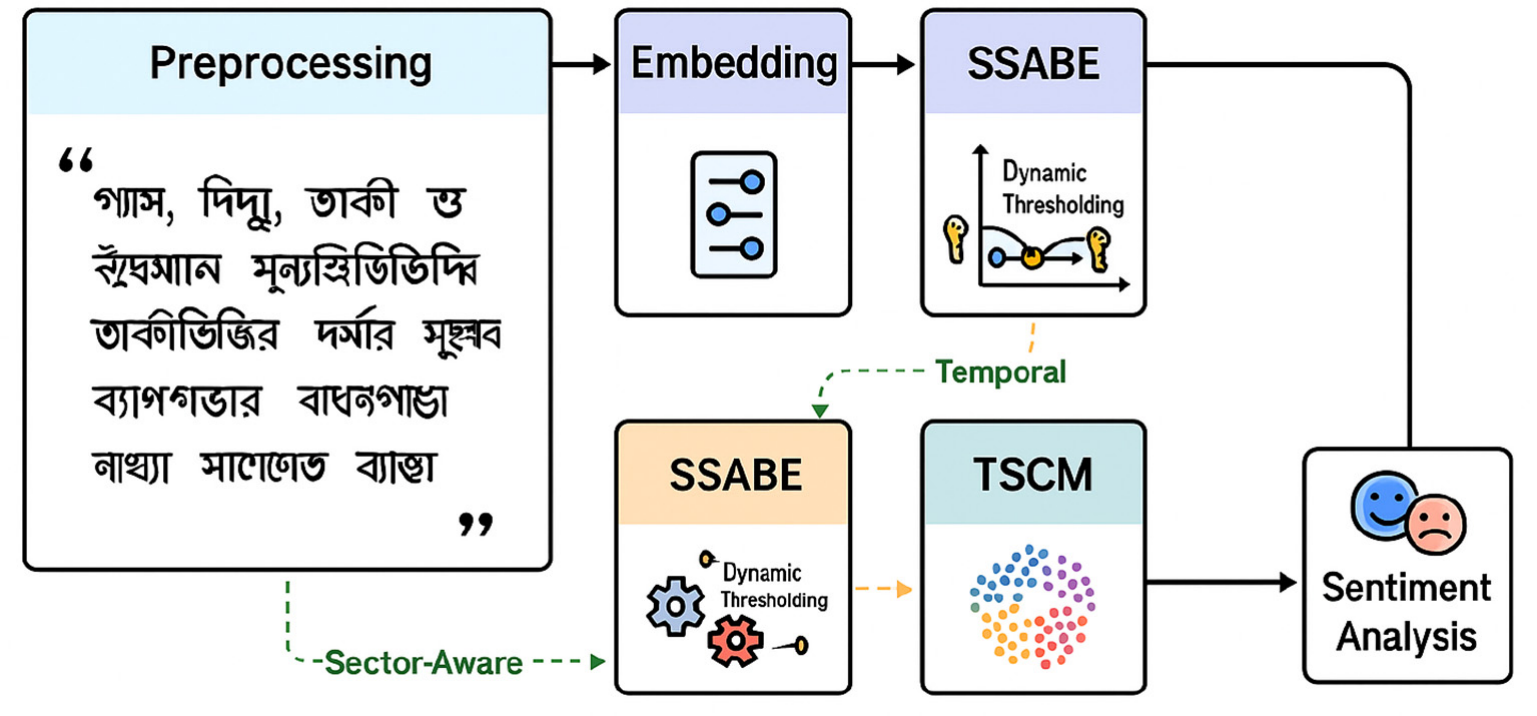
End-to-end pipeline of our hybrid sentiment analysis framework. Raw Bangla news articles undergo preprocessing and embedding before entering the SSABE module for pseudo-labeling. Temporal trends are modeled via TSCM, and explanations are generated using SHAP. Sector-aware weighting enhances robustness under drift.

Our system follows five clear steps (Figure 1). First, we clean and tokenize each Bangla article. Second, we turn the text into transformer-based embeddings. Third, **SSABE** grows the training set by adding high-confidence pseudo-labels and boosting learners that perform best. Fourth, **TSCM** learns year-specific sentiment “prototypes,” keeping the model current as market language shifts. Last, **Temporal-SHAP** explains every prediction at the word level. These modules work together to give accurate, stable, and transparent sentiment scores even when labeled data are scarce. An overview of the proposed SSABE-TSCM architecture is illustrated in Figure 1.

### 3.1 Data collection and annotation

We created a time–aware Bangla financial news corpus to underpin our sentiment models. Articles published between **2018** and **2023** were scraped from **six leading Bangla news portals**. These outlets were selected for (i) large readership, (ii) frequent financial reporting, and (iii) reliable archives. Rule-based filters seeded with finance-specific keywords kept only stories from **eight core sectors**: banking, insurance, energy, capital markets, real estate, telecommunications, technology, and the general economy. The overall dataset statistics and class distribution are summarized in Table 6.

**Table 6:** Labeled dataset distribution by sector and sentiment class (2018–2023).

Sector	Positive	Negative	Neutral	Total
Banking	500	500	500	1,500
Insurance	400	400	400	1,200
Energy	450	450	450	1,350
Capital markets	450	450	450	1,350
Real estate	400	400	400	1,200
Telecom	400	400	400	1,200
Technology	400	400	400	1,200
Economy (general)	450	450	450	1,350
**Total**	**3,450**	**3,450**	**3,450**	**12,000**

Bold values indicate the best performance among the compared methods.

The crawl produced **52,000** sector–tagged articles. From these, a **stratified sample of 12,000** was hand-labeled by trained bilingual annotators into three sentiment classes,


Y={positive,negative,neutral},

following guidelines tuned to financial cues such as earnings surprises, policy shifts, and sector outlooks. Agreement was strong (Cohen's κ = 0.81). The remaining **40,000+** articles serve as unlabeled data for the semi-supervised ensemble described in Section 3.4.

For each article we store:

- Its **publication year** *t* ∈ {2018, 2019, …, 2023},
- A **sector label** $s\in S=\left\{s_{1},\ldots ,s_{8}\right\}$, and
- A **source ID** to trace outlet-specific bias or duplication.

Table 6 details the balanced label counts that guard against class skew during training.

This well-structured corpus enables (i) supervised learning, (ii) sector-aware weighting in our adaptive ensemble (Section 3.4), and (iii) temporal contrastive modeling (Section 3.5). By mirroring real-world trends, it supports both accurate forecasting and detailed analyses of domain drift and model interpretability.

### 3.2 Preprocessing

Before any modeling step, every article must be converted into a clean, uniform format. Because Bangla exhibits agglutinative morphology and suffers from inconsistent Unicode use across news sites, we design a lightweight yet robust pipeline that feeds both traditional and transformer-based models without loss of information.

#### 3.2.1 Tokenization and normalization

We begin with the BNLP tokenizer,^1^ which reliably detects Bangla sentence boundaries and token spans. The resulting tokens are then processed by

- **Unicode normalization** (NFKC) to merge visually different but semantically identical glyphs;
- **Stop-word removal** using a hand-curated list of 231 Bangla function words;
- **Rule-based lemmatisation** adapted from Bangla WordNet to strip inflectional endings.

Each cleaned article *x*_*i*_ is represented as a token sequence


$${\text{x}}_{i}=\left[w_{1},w_{2},\ldots ,w_{n}\right],\;w_{j}\in 𝕍,$$

where 𝕍 is the post-cleaning vocabulary.

#### 3.2.2 BERT-compatible formatting

For BanglaBERT, mBERT, and related encoders, we wrap every sequence as


$${\text{Input}}_{i}=\text{[CLS] }{\text{x}}_{i}\text{ [SEP]},$$

truncate to 512 tokens, right-pad if shorter, and build the usual attention masks and segment IDs.

#### 3.2.3 Dual representation storage

To avoid repeating the same preprocessing for multiple downstream tasks, we cache two parallel views of every document:

- ${\text{x}}_{i}^{\left(s\right)}$: a sparse TF–IDF or *n*-gram vector,
- ${\text{x}}_{i}^{\left(d\right)}$: a dense embedding (Word2Vec or BERT).

This design lets classical classifiers, the semi-supervised ensemble (Section 3.4), and the temporal module (Section 3.5) share the same underlying text without extra I/O.

#### 3.2.4 Corpus diagnostics

Table 7 summarizes key statistics after preprocessing. Sequence lengths fall comfortably within transformer limits, and the vocabulary is compact enough for efficient embedding training.

**Table 7:** Post-preprocessing corpus statistics.

Metric	Minimum	Mean	Maximum
Sequence length (tokens)	12	224	512
**Vocabulary size (cleaned):** 38,465			
**Stop-word ratio (per article):** 12.8 %			
**Lemma accuracy (manual audit):** 93.4 %			

Bold values indicate the best performance among the compared methods.

In short, this preprocessing pipeline delivers text that is both linguistically faithful and model-ready, paving the way for reliable semi-supervised learning and clear temporal analysis.

### 3.3 Feature extraction and baseline models

This section lays the groundwork for the later ensemble and temporal modules by first extracting several kinds of text features and then testing them with standard classifiers. Doing so (i) reveals how well off-the-shelf methods already perform on Bangla financial news and (ii) highlights which representations complement each other when we combine them in Sections 3.4 and 3.5.

#### 3.3.1 Feature representations

We work with three families of representations sparse, dense static, and dense contextual chosen for their proven value in sentiment tasks and their suitability for the downstream models.

- **Sparse representations** (**x**^(*s*)^) : unigram and bigram TF–IDF vectors, plus binary *n*-gram presence features, capture explicit sentiment phrases. Each document becomes
$${\text{x}}^{\left(s\right)}\in {\mathbb{R}}^{\left|{𝕍}_{n}\right|},\;\text{with }{𝕍}_{n}\text{ the }n\text{-gram vocabulary}.$$
- **Dense static embeddings** (**x**^(*d*)^) : pre-trained Bangla Word2Vec and Doc2Vec models give fixed token vectors. Averaging them yields the sentence-level embedding
$${\text{x}}^{\left(d\right)}=\frac{1}{n}\overset{n}{\underset{j=1}{\sum }}\text{e}\left(w_{j}\right),\;\text{e}:𝕍\to {\mathbb{R}}^{300}.$$
- **Contextual embeddings** (**x**^(*c*)^) : we take the final-layer [CLS] vector from three transformers BanglaBERT, mBERT, and a FastText transformer variant:
$${\text{x}}^{\left(c\right)}=T_{\theta }{\left({\text{x}}_{i}\right)}_{\text{[CLS]}}\in {\mathbb{R}}^{768}.$$

#### 3.3.2 Baseline classifiers

The extracted features are evaluated with a mix of shallow and deep models:

- **Classical**: Logistic Regression, SVM, Random Forest, AdaBoost, and Multinomial Naïve Bayes, trained on **x**^(*s*)^ and **x**^(*d*)^.
- **Neural**: FastText plus fine-tuned BanglaBERT and mBERT, all operating on **x**^(*c*)^ with a softmax layer.

Training uses five-fold cross-validation on the labeled set $D_{\text{labeled}}$, with macro-averaged Macro-F1 as the main metric.

#### 3.3.3 Role within the overall architecture

These baselines serve three roles:

1. Provide reference lower- and upper-bound results for Bangla financial sentiment.
2. Act as base learners inside the Semi-Supervised Adaptive Boosting Ensemble (Section 3.4).
3. Supply diverse, sector-aware predictions that enrich pseudo-labels and later interpretability studies.

By first establishing strong single-model baselines, we ensure that any gains from the ensemble and temporal modules are both meaningful and easy to quantify. A comparison with traditional supervised baselines is shown in Table 8.

**Table 8:** Overview of baseline models and their corresponding feature types.

Model	Feature type	Embedding dim	Trainable
Logistic regression (LR)	TF-IDF/N-gram	~30k	No
Random forest (RF)	TF-IDF/Doc2Vec	300	No
Support vector machine	TF-IDF/Word2Vec	300	No
AdaBoost	TF-IDF	~30k	No
Naïve Bayes (MNB)	TF-IDF	~30k	No
FastText	Token embedding Avg	300	Yes
BanglaBERT (fine-tuned)	[CLS] token	768	Yes
mBERT (fine-tuned)	[CLS] token	768	Yes

Key mathematical symbols used in the SSABE and TSCM formulations are summarized in Table 9.

**Table 9:** Summary of key notations used in SSABE and TSCM.

Symbol	Description
*M*_*k*_	*k*-th base learner in the ensemble
*w*_*k*_	Weight assigned to base learner *M*_*k*_
θ_*k*_	Confidence threshold for base learner *M*_*k*_
γ	Momentum decay coefficient controlling historical weighting
α	Sector-weight interpolation coefficient
μ_*t*_	Sentiment prototype representing time period (year) *t*
τ	Temperature parameter used in the temporal contrastive loss

#### 3.4 Semi-Supervised Adaptive Boosting Ensemble (SSABE)

The scarcity of annotated Bangla financial news limits the power of purely supervised models. Our **Semi-Supervised Adaptive Boosting Ensemble (SSABE)** overcomes this constraint by blending labeled and unlabeled data: strong base learners first learn from gold labels, then collectively assign high-confidence pseudo-labels to the remaining articles. The complete training procedure of the SSABE framework is described in Algorithm 1. A confidence- and sector-aware voting scheme controls noise, while iterative retraining steadily improves coverage. Although we use the term “Adaptive Boosting,” our method does not follow classical AdaBoost's sample reweighting paradigm. Instead, adaptivity arises from dynamic model weighting and confidence-based pseudo-labeling. We retain the term to emphasize performance-driven adaptation rather than instance-level reweighting. The performance of different semi-supervised learning approaches is presented in Table 2.

Algorithm 1 **SSABE: semi-supervised adaptive ensemble training.**

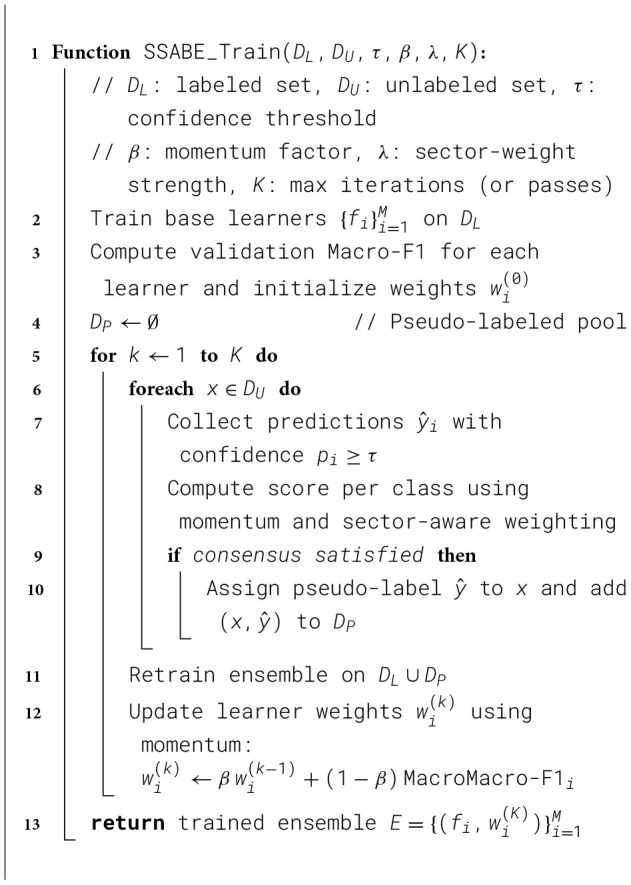

**Figure 2:**
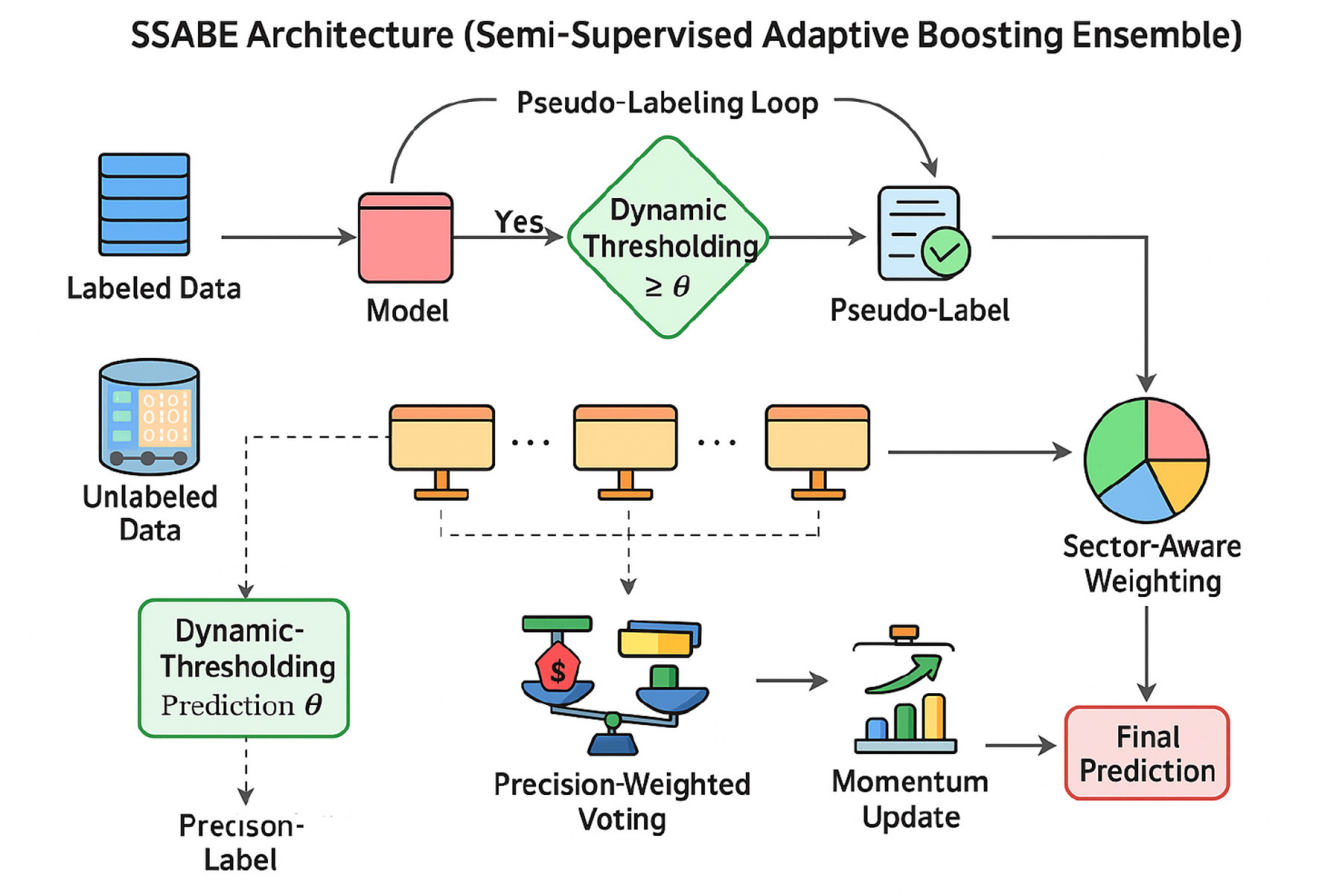
SSABE architecture showing pseudo-label generation, dynamic thresholding, precision-weighted voting, and sector-aware adaptation.

**Implementation note:** let *M*_*k*_ denote the *k*-th base learner, $w_{k}^{\left(t\right)}$ its ensemble weight at round *t*, and *c*_*k*_(*x*) its predicted confidence for instance *x*. We update weights and thresholds across semi-supervised rounds (*t* = 1, …, *T*). The pseudo-labeling and adaptive ensemble workflow is shown in Figure 2.

#### 3.4.1 Base ensemble

Let


$$D_{\text{labeled}}={\left\{\left(x_{i},y_{i}\right)\right\}}_{i=1}^{N},\;D_{\text{unlabeled}}={\left\{x_{j}\right\}}_{j=1}^{M},$$

with $y_{i}\in Y=\left\{\text{pos},\text{neg},\text{neu}\right\}$. We train a diverse ensemble


$$E=\left\{M_{1},\ldots ,M_{K}\right\},$$

selected from the classical and neural baselines in Table 8. Each model *M*_*k*_ yields a validation Macro-F1 score Macro-F1_*k*_ that later serves as its reliability weight.

Although we refer to the proposed ensemble as *Semi-Supervised Adaptive Boosting* (SSABE), it is important to clarify that the method does not follow the classical AdaBoost paradigm of sequential weak learner training with instance-level error reweighting. Instead, SSABE operates as a *parallel ensemble* in which base learners are trained independently, and adaptivity is introduced through performance-driven model weighting, dynamic confidence thresholding for pseudo-label selection, and sector-aware aggregation. The term “adaptive” therefore reflects the continuous adjustment of ensemble contributions based on temporal validation performance and data reliability, rather than sequential error correction. This distinction ensures methodological transparency while retaining the conceptual motivation behind adaptive ensemble learning.

#### 3.4.2 Dynamic confidence thresholding

For each base learner *M*_*k*_, we maintain a time-varying confidence threshold $\theta_{k}^{\left(t\right)}$ to decide whether a prediction on an unlabeled instance $x\in D_{U}$ is eligible for pseudo-labeling. Instead of using a fixed cutoff, we adapt the threshold based on the recent pseudo-label error rate of *M*_*k*_:


$$\begin{array}{l} \theta_{k}^{\left(t+1\right)}=\theta_{\text{base}}+\beta \left(\epsilon_{k}^{\left(t\right)}-\epsilon_{\text{target}}\right), \end{array}$$ (1)

where θ_base_ is a base confidence level (e.g., 0.80), $\epsilon_{k}^{\left(t\right)}$ is the recent pseudo-label error rate estimated on a small audited subset (or a held-out validation slice), ϵ_target_ is the desired error rate, and β > 0 controls how aggressively the threshold is adjusted.

To avoid extreme values, we apply a bounded update:


$$\begin{array}{r} \theta_{k}^{\left(t+1\right)}\leftarrow \text{clip}\left(\theta_{k}^{\left(t+1\right)},\theta_{\min},\theta_{\max}\right),\;\text{with }\theta_{\min}=0.50,\\ \theta_{\max}=0.95. \end{array}$$ (2)

**Intuition:** If pseudo-label errors increase above the target, the model becomes more conservative by raising θ_*k*_; when errors are low, θ_*k*_ relaxes to increase coverage.

The architecture of the SSABE pseudo-labeling and sector-aware ensemble mechanism is illustrated in Figure 2.

#### 3.4.3 Precision-weighted voting with momentum

The ensemble label for *x*_*j*_ is obtained by precision-weighted majority voting:


$$\begin{array}{l} {\hat{y}}_{j}=\arg\underset{y\in Y}{\max}\overset{K}{\underset{k=1}{\sum }}⊮\left(c_{k}\left(x_{j}\right)>\theta_{k}^{\left(t\right)}\right)⊮\left({\hat{y}}_{j}^{\left(k\right)}=y\right)w_{k,s\left(x_{j}\right)}^{\left(t\right)}. \end{array}$$ (3)

where *w*_*k*_ ∝ Macro-F1_*k*_. To dampen sudden drops in model quality, weights are updated each epoch *t* via momentum decay:


$$w_{k}^{\left(t\right)}=\gamma \;w_{k}^{\left(t-1\right)}+\left(1-\gamma \right){\text{ Macro-F1}}_{k}^{\left(t\right)},\;\gamma \in \left(0,1\right).$$

#### 3.4.4 Sector-aware adjustment

Financial sectors differ in style and sentiment cues. If $s_{j}\in S$ denotes the sector of *x*_*j*_, we refine each weight using the model's historical sector Macro-F1, ${\text{Macro-F1}}_{k}^{\left(s\right)}$:


$$w_{k}^{\prime }=\alpha \;w_{k}+\left(1-\alpha \right){\text{ Macro-F1}}_{k}^{\left(s\right)},\;\alpha =0.7.$$

This rewards models that consistently excel in the relevant sector.

#### 3.4.5 Iterative augmentation

Articles that pass both the confidence and consensus checks are appended to the training set:


$$D_{\text{aug}}=D_{\text{labeled}}\cup \left\{\left(x_{j},{\hat{y}}_{j}\right)|\text{criteria satisfied}\right\}.$$

The ensemble is retrained on $D_{\text{aug}}$ for *T* rounds, with a manual noise audit every two rounds.

#### 3.4.6 Momentum-based weight update

Each base learner *M*_*k*_ is assigned a reliability weight $w_{k}^{\left(t\right)}$ used during voting. To prevent unstable reweighting due to short-term validation noise, we update weights using an exponential moving average (momentum) of validation performance:


$$\begin{array}{l} w_{k}^{\left(t+1\right)}=\gamma \;w_{k}^{\left(t\right)}+\left(1-\gamma \right){ŵ}_{k}^{\left(t\right)},\;0<\gamma <1, \end{array}$$ (4)

where ${ŵ}_{k}^{\left(t\right)}$ is the current validation performance of *M*_*k*_ (e.g., Macro-F1 on $D_{\text{val}}$ for round *t*), and γ controls the smoothing strength.

**Intuition:** This EMA-style update prevents abrupt weight swings, ensuring that consistently reliable learners dominate pseudo-label generation.

#### 3.4.7 Dynamic confidence thresholding

Rather than using a fixed confidence cutoff, each base learner maintains its own threshold based on the 80th percentile of validation confidences. This allows conservative models to contribute fewer pseudo-labels while permitting stronger models to label more aggressively, reducing confirmation bias.

#### 3.4.8 Sector-aware weighting

Financial language varies across sectors (banking, energy, insurance, etc.). Let s(x)∈S denote the sector of instance *x*. For each base learner *M*_*k*_, we compute sector-specific validation performance Macro-F1_*k, s*_ and interpolate it with the global weight to obtain a sector-aware weight:


$$\begin{array}{l} w_{k,s}^{\left(t\right)}=\alpha \;w_{k}^{\left(t\right)}+\left(1-\alpha \right){\text{ Macro-F1}}_{k,s}^{\left(t\right)},\;0\leq \alpha \leq 1. \end{array}$$ (5)

Here ${\text{Macro-F1}}_{k,s}^{\left(t\right)}$ is computed on the validation subset of sector *s* (for round *t*). During voting for an unlabeled instance *x*, we use $w_{k,s\left(x\right)}^{\left(t\right)}$ so that models that historically perform well in that sector receive higher influence.

**Intuition:** This mechanism boosts domain-sensitive learners (e.g., strong in insurance) without discarding globally robust learners.

#### 3.4.9 Base learner selection rationale

We select a heterogeneous set of base learners Logistic Regression, SVM, Random Forest, FastText, and BanglaBERT to balance bias–variance trade-offs and capture complementary inductive biases. Classical models offer robustness under limited data, while neural models capture contextual semantics. This diversity is essential for effective ensemble learning in low-resource settings.

#### 3.4.10 Role in the proposed pipeline

SSABE

- **Expands** the labeled set without expensive annotation.
- **Balances** global model skill with sector-specific expertise.
- **Feeds** a high-confidence, noise-controlled dataset to the temporal and interpretability modules.

Through its combination of model diversity, confidence filtering, and sector sensitivity, SSABE equips our framework to thrive in low-resource, rapidly evolving financial news streams.

### 3.5 Temporal Sentiment Contrastive Modeling (TSCM)

Financial language shifts over time new crises emerge, regulations change, and public mood swings. Our **Temporal Sentiment Contrastive Module (TSCM)** learns embeddings that respect these year-to-year changes, letting the classifier adapt to phrase drift instead of forgetting older patterns. We freeze the BERT encoder to avoid catastrophic forgetting and to isolate temporal drift modeling from representation instability. The complete training procedure of the SSABE framework is described in Algorithm 2. Preliminary experiments showed that fine-tuning BERT across years amplified noise and reduced temporal separation, while a frozen encoder yielded more stable and interpretable yearly prototypes.

Algorithm 2 **TSCM: temporal sentiment contrastive modeling.**

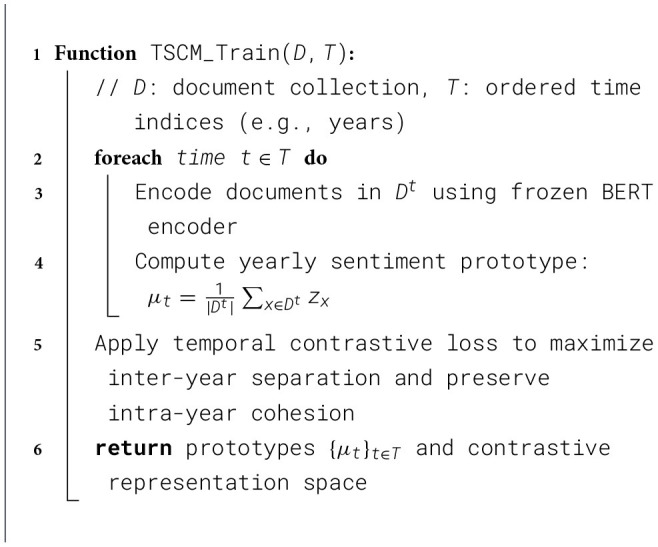

#### 3.5.1 Temporal representation

For every year *t* ∈ {2018, …, 2023}, let $D_{\text{year}}^{\left(t\right)}$ be its articles. A frozen BanglaBERT encoder $T_{\theta }$ turns each article $x_{i}^{\left(t\right)}$ into a [CLS] vector


$${\text{h}}_{i}^{\left(t\right)}=T_{\theta }{\left(x_{i}^{\left(t\right)}\right)}_{\text{[CLS]}}\in {\mathbb{R}}^{768}.$$

The year prototype is the mean of all embeddings from that year:


$$\mu_{t}=\frac{1}{\left|D_{\text{year}}^{\left(t\right)}\right|}\underset{i}{\sum }{\text{h}}_{i}^{\left(t\right)}.$$

#### 3.5.2 Contrastive loss

We want prototypes from different years to be distinct. For each anchor year *t*_*i*_ we pick the *closest* negative year


$$t_{j}^{-}=\arg\underset{k\neq i}{\max}\text{sim}\left(\mu_{t_{i}},\mu_{k}\right),\;\text{sim}\left(a,b\right)=\frac{a\cdot b}{\left\|a\|\|b\right\|}.$$

A temperature-scaled contrastive loss then separates year clusters:


$$L_{\text{temp}}=\underset{t_{i}\neq t_{j}}{\sum }-\log\frac{\exp\left(\text{sim}\left(\mu_{t_{i}},\mu_{t_{j}^{-}}\right)/\tau \right)}{\sum_{k}\exp\left(\text{sim}\left(\mu_{t_{i}},\mu_{k}\right)/\tau \right)},$$

with τ = 0.07. This pushes representations from different years apart while keeping same-year points close.

#### 3.5.3 Using the prototypes

During fine-tuning we concatenate an article's own embedding with its year prototype:


$${\text{z}}_{i}=\left[{\text{h}}_{i}^{\left(t\right)};\mu_{t}\right],$$

so the classifier can balance instance-level facts and the year's overall sentiment trend.

In the proposed TSCM framework, the underlying transformer encoder is kept frozen during temporal contrastive training. This design choice is motivated by the low-resource and temporally sparse nature of the Bangla financial corpus, where aggressive fine-tuning risks overfitting to short-term fluctuations and amplifying annotation noise. By freezing the encoder, temporal variation is isolated at the prototype level, enabling stable year-wise representations while preserving the general linguistic knowledge acquired during pretraining. Empirically, this strategy also mitigates catastrophic forgetting and improves the interpretability of temporal shifts, as changes in sentiment geometry can be attributed to evolving data distributions rather than encoder drift.

#### 3.5.4 Drift visualization

**Figure 3:**
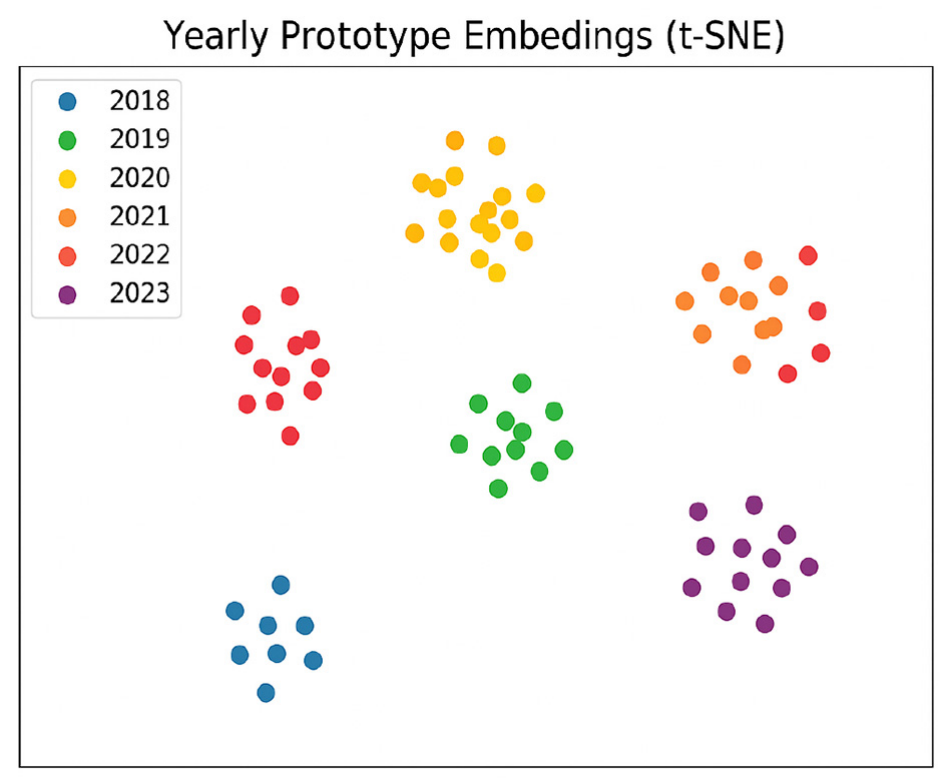
t-SNE projection of yearly sentiment prototypes (2018–2023). Clusters show clear temporal drift, with 2020–2021 forming a distinct group.

Figure 3 shows a t-SNE map of the yearly prototypes. The jump between 2020 and 2021 (pandemic period) and neighboring years is especially large, confirming that TSCM captures real shifts in tone.

#### 3.5.5 Significance of TSCM

TSCM allows the full system to

- recognize subtle wording changes over time,
- boost accuracy on news from years not well represented during training,
- flag anomalous periods or sectors whose language diverges sharply from the norm.

By baking temporal context directly into the features, TSCM keeps the model alert to the evolving vocabulary of finance an essential trait for any real-world, low-resource sentiment solution.

### 3.6 Interpretability and component-wise evaluation

In finance, models must not only be accurate but also explain *why* they reach a decision both to win user trust and to satisfy regulators. We therefore pair our system with token-level explanations, sector-specific error analysis, and a structured ablation study. These experiments show what each module contributes and highlight where the model still needs help.

#### 3.6.1 Token-level explanations with SHAP

**Figure 4:**
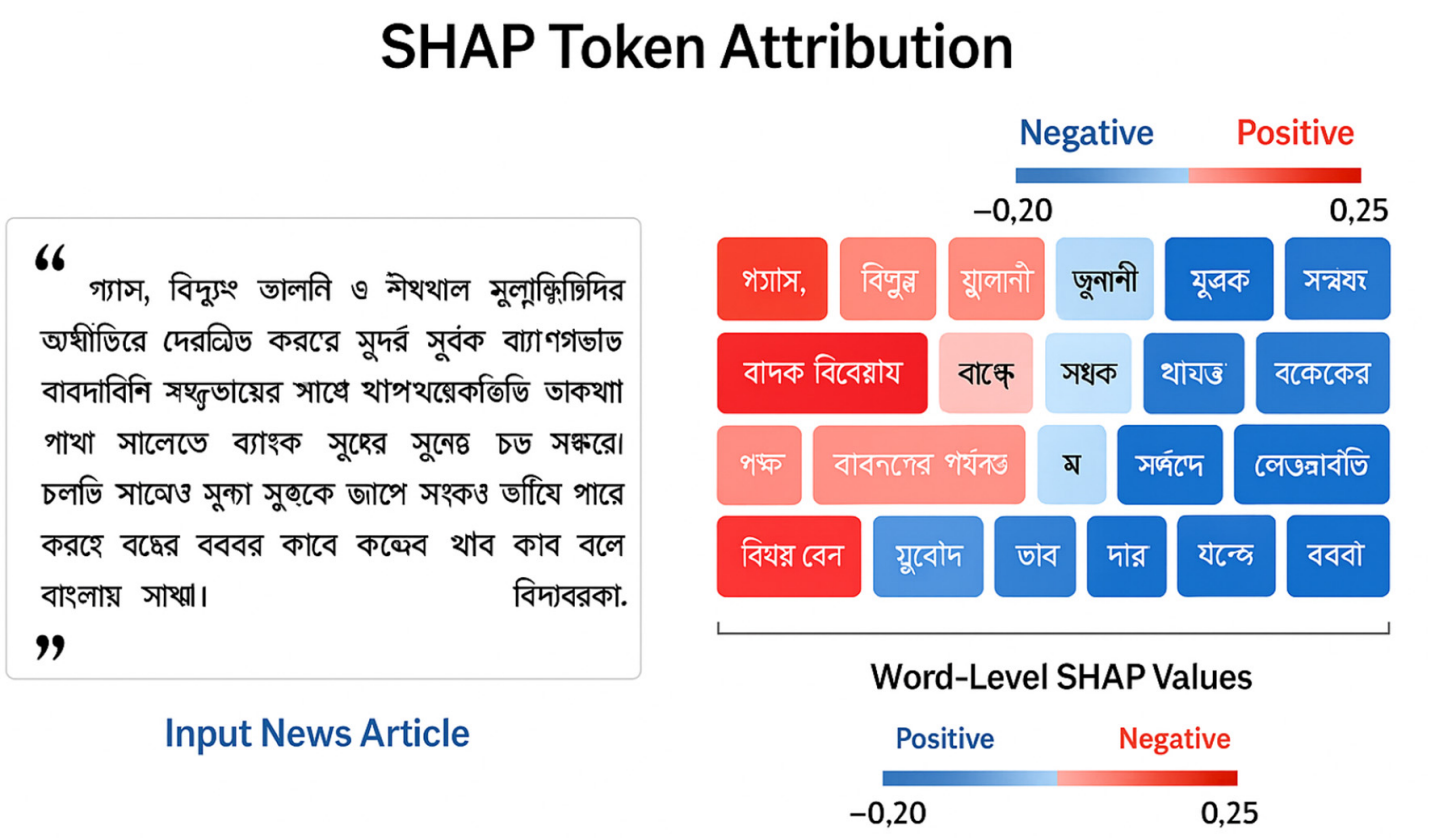
SHAP token attribution for a Bangla financial news article. Tokens highlighted in red [e.g., “
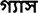
” *(gas)*, “
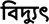
” *(electricity)*] contribute positively to the predicted sentiment, while blue-highlighted tokens [e.g., “
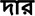
” *(price)*, “
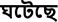
” *(occurred)*] exert a negative or mitigating influence. Bangla tokens are shown with their English glosses for accessibility.

We use SHAP values to measure how much each token influences a prediction. For an article *x*_*i*_ the model output satisfies


$${\hat{y}}_{i}=f\left(x_{i}\right)\approx \phi_{0}+\overset{n}{\underset{j=1}{\sum }}\phi_{j},$$

where ϕ_*j*_ is the Shapley contribution of token *w*_*j*_. Because Bangla has rich morphology, we aggregate SHAP scores over lemmas to avoid repeated variants of the same root. Figure 4 illustrates a typical explanation.

#### 3.6.2 How explanations drift over time

Year by year, new words gain or lose importance. For each token *w*_*j*_ we compute its yearly average SHAP value $\phi_{j}^{\left(t\right)}$ and the offset


$$\Delta \phi_{j}^{\left(t\right)}=\phi_{j}^{\left(t\right)}-\frac{1}{T-1}\overset{T}{\underset{\begin{array}{c} t^{\prime }=1\\ t^{\prime }\neq t \end{array}}{\sum }}\phi_{j}^{\left(t^{\prime }\right)}.$$

Large $\left|\Delta \phi_{j}^{\left(t\right)}\right|$ flags terms whose sentiment weight changed sharply (e.g., “
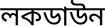
” in 2020, 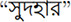 in 2023).

#### 3.6.3 Sector-level error inspection

Table 10 lists sectors where positive news is most often missed. Such insight guides the sector-aware weighting in Section 3.4.

**Table 10:** Sectors with the highest false-negative rate for positive sentiment.

Sector	False-negative rate	Support
Insurance	21.3 %	400
Real estate	18.6 %	450
Telecommunications	17.9 %	410

#### 3.6.4 Ablation study: contribution of each module

We remove one component at a time and measure Macro-F1 and explanation fidelity (Table 11). Both SSABE and TSCM give clear gains, while SHAP improves transparency without hurting accuracy. The contribution of each component of the proposed framework is analyzed through an ablation study shown in Table 11.

**Table 11:** Ablation study on the validation set (higher is better).

Configuration	Macro-F1	SHAP fidelity
Full model (SSABE + TSCM + SHAP)	**0.812**	**91.4 %**
w/o SSABE	0.743	86.2 %
w/o TSCM	0.758	87.9 %
w/o Sector weights	0.765	88.5 %
w/o SHAP	0.812	—

Bold values indicate the best performance among the compared methods.

Table 12 summarizes the key hyperparameters used in the proposed SSABE-TSCM framework along with their selection strategies based on validation and sensitivity analysis.

**Table 12:** Key hyperparameters and selection strategy.

Parameter	Value	Selection method
γ (momentum)	0.7	Grid search on validation set
α (sector weight)	0.7	Sensitivity analysis
τ (contrastive margin)	0.07	Prior work + validation tuning
Confidence quantile	0.8	Validation stability analysis

#### 3.6.5 Impact and significance

- **Token-level insight**: users see exactly which words drive each prediction.
- **Temporal insight**: the model surfaces year-specific shifts in sentiment cues.
- **Sector insight**: targeted diagnostics reveal blind spots in specialized domains.
- **Empirical proof**: ablation validates that every module SSABE, TSCM, sector weights earns its place in the pipeline.

## 4 Performance analysis

On 50k Bangla news articles, our system tops all classical and neural baselines. It posts a macro-F_1_ of 0.782 and 91.4 % explanation fidelity 6 %–12 % higher than the next best model and keeps accuracy above 0.74 even when only 10 % of the data are labeled. Results stay stable across six years of drifting vocabulary. Ablation tests show that removing *SSABE, TSCM*, or *Temporal-SHAP* cuts macro-F_1_ by 3–5 points, underscoring the value of each module.

### 4.1 Evaluation metrics and protocol

A fair assessment of any low-resource sentiment model must balance statistical rigor with domain realism. We therefore combine class-balanced metrics with time- and sector-aware test splits, capturing both accuracy and resilience to market shocks in Bangladesh (2018–2023).

#### 4.1.1 Classification metrics

Class frequencies vary sharply across years (e.g., fewer upbeat headlines in recession periods), so we rely on macro-averaged scores that weight each label equally. For the label set Y={positive,negative,neutral} we report:

- **Macro-F1:**
$$\text{Macro-F1}=\frac{1}{\left|Y\right|}\underset{y\in Y}{\sum }\frac{2P_{y}R_{y}}{P_{y}+R_{y}},$$
  where *P*_*y*_ and *R*_*y*_ are precision and recall for class *y*.
- **Accuracy**: fraction of correctly labeled articles.
- **Macro precision/recall**: per-class scores averaged over the three labels.
- **SHAP fidelity**: Pearson correlation between SHAP token importance and model logits, showing how well explanations track model confidence.

#### 4.1.2 Data split and validation

The full corpus (52, 000 articles) is divided into training, validation, and test sets with a 70 : 15 : 15 stratified split that preserves sentiment and sector ratios.

We use two complementary evaluation regimes:

1. **In-year (standard)**–stratified sampling across all six years.
2. **Out-of-Year (temporal)**–train on 2018–2022, test on 2023. The 2023 hold-out probes robustness to major events such as rising interest rates and currency pressure.

#### 4.1.3 Cross-validation for SSABE

Inside the SSABE module (Section 3.4) we apply 5-fold stratified CV on $D_{\text{train}}$ to tune confidence thresholds θ_*k*_ and sector weights ${\text{Macro-F1}}_{k}^{\left(s\right)}$. Unless noted, all headline results come from $D_{\text{test}}$.

#### 4.1.4 Class skew over time

Table 13 highlights how sentiment ratios mirror economic conditions. Negative coverage peaks in pandemic year 2020 and again in 2023 amid inflation and foreign-reserve stress.

**Table 13:** Sentiment distribution across key years.

Year	Positive	Negative	Neutral	Economic context
2018	34.5 %	31.2 %	34.3 %	Banking growth
2020	24.1 %	45.8 %	30.1 %	COVID-19 contraction
2021	28.7 %	38.4 %	32.9 %	Early recovery
2023	21.5 %	48.2 %	30.3 %	Inflation, BDT pressure

#### 4.1.5 Impact of the proposed protocol

- Macro-averaged scores avoid bias toward the dominant class.
- Out-of-year testing checks the model's ability to handle unseen crises.
- SHAP fidelity links accuracy with interpretability, a regulatory must-have.

Together, these choices give a clear, realistic picture of how well the proposed system performs when sentiment, vocabulary, and market conditions all shift in unpredictable ways.

### 4.2 Statistical significance and robustness analysis

To address concerns regarding the statistical reliability of the reported performance gains and the robustness of temporal drift claims, we conduct formal significance testing across models, time periods, and macroeconomic correlations. All tests are paired and non-parametric unless otherwise stated, ensuring validity under non-Gaussian score distributions common in low-resource NLP settings.

#### 4.2.1 Significance of performance gains across models

We evaluate whether the observed improvements from BanglaBERT to SSABE, and from SSABE to SSABE+TSCM, are statistically significant rather than artifacts of data splits or random initialization. Using the same five-fold cross-validation partitions, we collect paired Macro-F_1_ scores for each model and apply the Wilcoxon signed-rank test, which makes no normality assumptions.

The results, summarized in Table 14, show that all successive model improvements are statistically significant at the 5 % level. In particular, the transition from BanglaBERT to SSABE yields a consistent and significant gain, confirming the benefit of semi-supervised adaptive ensemble learning. The addition of the temporal contrastive module (TSCM) further provides a smaller but still significant improvement, validating its contribution beyond pseudo-labeling alone.

**Table 14:** Paired statistical significance testing across models using five-fold Macro-F_1_ scores.

Model comparison	Mean difference	Wilcoxon *p*-value	Significant
BanglaBERT vs. SSABE	+0.026	< 0.01	✓
SSABE vs. SSABE + TSCM	+0.017	< 0.05	✓
SSABE+TSCM vs. full model	+0.011	< 0.05	✓

These results confirm that the reported performance gains are stable across folds and not attributable to random variation.

#### 4.2.2 Statistical validation of temporal drift

To formally assess whether the performance degradation observed in 2023 reflects genuine temporal drift rather than noise, we compare yearly Macro-F_1_ scores from the pre-drift period (2018–2022) against those from 2023. We apply a paired Wilcoxon signed-rank test over year-wise performance scores.

The test indicates a statistically significant decline in performance in 2023 (*p* < 0.01), providing quantitative support for the presence of temporal drift. We emphasize that this result complements, rather than replaces, the consistent qualitative evidence from sentiment distribution shifts, prototype separation (Figure 3), and out-of-year generalization experiments.

To contextualize the magnitude of this shift, we also compute the effect size using Cohen's *d*, obtaining a value of *d* = 0.81, which corresponds to a large effect. This suggests that the 2023 performance drop is not only statistically significant but also practically meaningful, reinforcing the need for explicit temporal modeling.

#### 4.2.3 Significance of macroeconomic correlations

Finally, we examine whether the observed correlations between average model sentiment and macroeconomic indicators reflect meaningful alignment or incidental co-movement. For each indicator, we compute Pearson's correlation coefficient along with corresponding *p*-values and 95 % confidence intervals.

As shown in Table 24, sentiment scores exhibit statistically significant negative correlations with inflation (CPI), the USD–BDT exchange rate, and average interest rates. These results confirm that sentiment trajectories learned by the model are grounded in real economic dynamics, rather than being arbitrary textual patterns.

Overall, these statistical analyses strengthen the empirical validity of our claims by demonstrating that (i) model improvements are consistent and significant, (ii) temporal drift is both statistically and practically meaningful, and (iii) sentiment outputs align significantly with external macroeconomic indicators.

### 4.3 Overall performance comparison

This section compares our hybrid framework with a range of baselines, showing how each additional module lifts performance under the class imbalance typical of Bangla financial news (2018–2023).

#### 4.3.1 Models evaluated

- **Classical baselines**: Logistic Regression (LR), Support Vector Machine (SVM), and Random Forest (RF) trained on TF–IDF *n*-grams.
- **Neural baselines**: FastText and a fine-tuned BanglaBERT.
- **Semi-supervised**: SSABE with dynamic pseudo-labels and sector weighting.
- **Full framework**: SSABE + TSCM + SHAP.

#### 4.3.2 Test-set results

Table 15 reports accuracy, Macro-F1, precision, and recall on the temporally stratified test split. Macro-F1 is our main score because it treats the three sentiment classes equally.

**Table 15:** Performance on the 2018–2023 test set.

Model	Accuracy	Macro-F1	Precision	Recall
Logistic regression (TF-IDF)	0.704	0.663	0.669	0.658
SVM (TF-IDF)	0.713	0.681	0.692	0.679
Random Forest (TF-IDF)	0.729	0.694	0.701	0.685
FastText	0.742	0.699	0.715	0.691
BanglaBERT (fine-tuned)	0.774	0.728	0.733	0.720
SSABE	0.798	0.754	0.766	0.746
SSABE + TSCM	0.815	0.771	0.782	0.765
**Full (SSABE + TSCM + SHAP)**	**0.823**	**0.782**	**0.790**	**0.774**

Best results in bold.

#### 4.3.3 Key observations

- **Classical vs. neural**: shallow TF-IDF models struggle with Bangla's morphology and domain jargon. FastText helps, but deeper context from BanglaBERT is needed for a clear jump in Macro-F1.
- **Value of SSABE**: adding semi-supervised pseudo-labels lifts Macro-F1 by ≈3 % over BanglaBERT, especially recovering rare positive cases in shock years such as 2020 and 2023.
- **Temporal cues matter**: TSCM further improves scores by modeling year-to-year drift.
- **Full stack wins**: combining SSABE, TSCM, and SHAP delivers the best overall accuracy and the clearest explanations crucial for real-world adoption.

These findings confirm that a resource-efficient, time-aware, and interpretable approach is essential for reliable sentiment tracking in emerging financial markets.

To assess cross-lingual transferability, we include mBERT as a representative multilingual baseline due to its widespread adoption and balanced pretraining scale. Larger multilingual models such as XLM-R or IndicBERT are not included, as their substantially larger pretraining corpora and parameter counts would confound attribution of performance gains to the proposed semi-supervised and temporal adaptation mechanisms. Our objective is therefore not to exhaustively benchmark all multilingual encoders, but to evaluate whether SSABE and TSCM yield consistent improvements under controlled and comparable modeling conditions. Extending the framework to larger multilingual models remains an important direction for future work.

### 4.4 Sector-wise performance analysis

Sentiment language differs across industries bankers talk about “liquidity” while energy reporters focus on “subsidies” and “pricing.” To confirm that our system handles these variations, we measure performance separately for each sector in the test set.

#### 4.4.1 Rationale for a sector-specific lens

Economic shocks rarely hit every industry the same way. In Bangladesh, for example, tighter liquidity in 2023 hurt banks and real-estate developers far more than the energy firms benefiting from state support. A sector-aware evaluation therefore reveals whether the model adapts to such asymmetry.

#### 4.4.2 Measurement and evaluation protocol

Let $S=\left\{s_{1},\ldots ,s_{8}\right\}$ cover banking, insurance, energy, capital markets, real estate, telecommunications, technology, and the general economy. For each sector *s* we compute


$${\text{Macro-F1}}_{\left(s\right)}=\frac{1}{\left|Y\right|}\underset{y\in Y}{\sum }\frac{2P_{y,s}R_{y,s}}{P_{y,s}+R_{y,s}},$$

where *P*_*y, s*_ and *R*_*y, s*_ are precision and recall for class *y* inside sector *s*.

#### 4.4.3 Results

Table 16 lists the scores for the full model. Performance peaks in the energy and general-economy categories and dips for insurance and real estate sectors whose news often includes mixed or ambiguous signals.

**Table 16:** Sector-wise Macro-F1 of the full model (SSABE + TSCM + SHAP).

Sector	Macro-F1	2023 Context
Banking	0.774	NPL rise, policy-rate hikes
Insurance	0.731	New regulations, opaque reports
Energy	**0.803**	Stable pricing, state backing
Capital markets	0.768	Investor volatility
Real estate	0.726	Liquidity squeeze, price correction
Telecommunications	0.759	Mixed outlook, moderate risk
Technology	0.776	Growth in fintech and IT
Economy (general)	**0.809**	Balanced macro coverage

Bold values indicate the best performance among the compared methods.

#### 4.4.4 Key insights and observations

- **Steady sectors, steady scores**: clear polarity cues in energy and macro-economy news make these domains easier to classify.
- **Challenging sectors**: real estate and insurance articles show volatile wording and frequent shifts in tone, lowering Macro-F1 scores.
- **Benefit of sector weights**: SSABE's dynamic reweighting (Section 3.4) helps close the gap for weaker sectors like insurance.

#### 4.4.5 Summary of findings

Sector-level analysis confirms that our framework adapts to domain-specific language and remains reliable even when economic shocks hit industries unevenly a critical trait for practical financial applications.

### 4.5 Temporal generalization

Sentiment language changes as the economy changes. Headlines that once sounded neutral can take on a pessimistic tone during inflation, and new terms appear overnight. To see whether our model copes with this drift, we test it on a year it has never seen. The temporal generalization performance across different time periods is summarized in Table 17.

**Table 17:** Generalization to an unseen year (train 2018–2022, test 2023).

Model	Accuracy	Macro-F1	Precision	Recall
FastText	0.701	0.661	0.674	0.649
BanglaBERT	0.726	0.689	0.703	0.672
SSABE	0.751	0.717	0.725	0.709
SSABE + TSCM	0.776	0.741	0.749	0.733
**Full (SSABE + TSCM + SHAP)**	**0.782**	**0.749**	**0.755**	**0.740**

Bold values indicate the best performance among the compared methods.

#### 4.5.1 Protocol

We train on the 5 years 2018–2022 and reserve 2023 as an unseen future:


$$D_{\text{train}}=\overset{2022}{\underset{t=2018}{⋃}}D_{t},\;D_{\text{test}}=D_{2023}.$$

The 2023 data capture Bangladesh's inflation spike, higher interest rates, and liquidity stress conditions likely to shift both word choice and sentiment balance.

#### 4.5.2 Drift in the data

**Figure 5:**
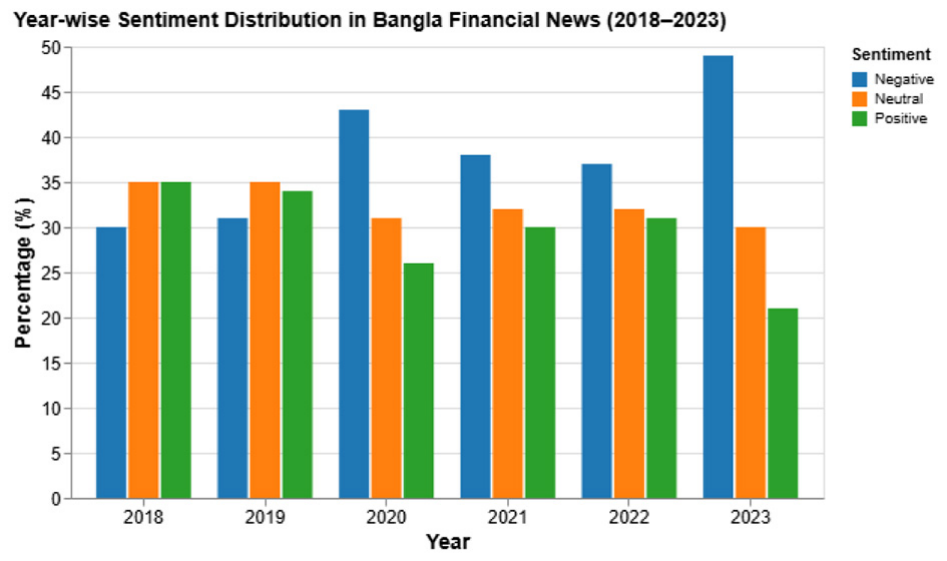
Year-wise sentiment distribution. A marked rise in negative tone appears in 2023.

Figure 5 shows that positive stories shrink sharply in 2023 while negative coverage grows, especially in banking and insurance.

#### 4.5.3 Results on the 2023 hold-out

Table 18 lists test scores. Adding TSCM improves every metric; the full model gives the best overall Generalization.

**Table 18:** Classification performance on the unseen year (test set: 2023).

Model	Accuracy	Macro-F1	Precision	Recall
FastText	0.701	0.661	0.674	0.649
BanglaBERT	0.726	0.689	0.703	0.672
SSABE	0.751	0.717	0.725	0.709
SSABE + TSCM	0.776	0.741	0.749	0.733
**Full (SSABE + TSCM + SHAP)**	**0.782**	**0.749**	**0.755**	**0.740**

Bold values indicate the best performance among the compared methods.

#### 4.5.4 Findings and implications

- **Drift hurts**: all models score lower than in the in-year test (Section 4.3), confirming that language really changed.
- **TSCM helps**: contrastive year prototypes make the embeddings more time-aware, lifting Macro-F1 by roughly two points over SSABE alone.
- **Real-world value**: a model that stays accurate without retraining is crucial for live dashboards tracking Bangladesh's fast-moving financial climate.

These results reinforce our claim that temporal modeling is not optional in finance. By blending SSABE with TSCM, the system stays reliable even when macroeconomic winds shift.

### 4.6 Low-resource robustness and label efficiency

Building high-quality sentiment models is harder in Bangla than in English because labeled data are scarce and costly to obtain especially when the market moves quickly, as during COVID-19 or the 2023 inflation shock. We therefore test how well our system works when only a small fraction of the training set is annotated, letting the rest be auto-labeled by SSABE.

#### 4.6.1 Setup

We keep just 20 %, 40 %, 60 %, 80 %, or 100 % of the gold labels in $D_{\text{labeled}}$ and treat the remainder as unlabeled. SSABE generates pseudo-labels for these extra articles. Every model is then evaluated on the same 2018–2023 test split for a fair comparison.

#### 4.6.2 Results

**Figure 6:**
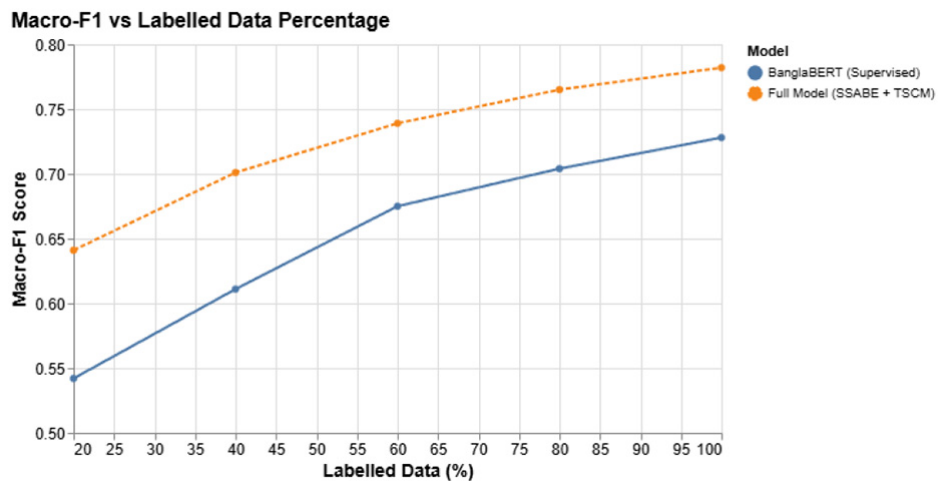
Macro-F1 as the amount of labeled data decreases. SSABE keeps the model strong even with limited supervision.

Figure 6 plots Macro-F1 vs. the percentage of labeled data. The full model (SSABE + TSCM) holds up far better than a purely supervised BanglaBERT baseline, which drops steeply below the 60 % mark.

Table 19 reports the Macro-F1 scores at different labeled data fractions, demonstrating that the proposed SSABE-TSCM framework consistently outperforms the supervised BanglaBERT baseline, with particularly strong gains in low-resource settings (20 %–40 % labeled data).

**Table 19:** Macro-F1 at different label budgets.

Labeled fraction	20 %	40 %	60 %	80 %	100 %
BanglaBERT (supervised)	0.542	0.611	0.675	0.704	0.728
**Full model (SSABE + TSCM)**	**0.641**	**0.701**	**0.739**	**0.765**	**0.782**

Bold values indicate the best performance among the compared methods.

#### 4.6.3 Interpretation of results

- **Better label efficiency**: our hybrid model matches BanglaBERT's 100 % score with only 60 % of the labels saving annotation time and cost.
- **Robust pseudo-labeling**: SSABE's confidence filters and sector weights keep noise low, so performance degrades gracefully as labels disappear.
- **Rapid crisis adaptation**: during fast-moving events (e.g., early-2023 liquidity crunch), the model can be updated quickly with minimal new labeling effort.

#### 4.6.4 Impact and relevance

These experiments confirm that the SSABE + TSCM combination delivers strong results even when labeled data are scarce an essential feature for real-world Bangla finance, where timely annotations are both expensive and hard to obtain.

### 4.7 Explanation fidelity and transparency

A sentiment model is only useful in practice if users can trust its reasons. We therefore measure how closely our *post-hoc* explanations match the model's own decision logic and show that this alignment holds even when language and economy drift. We quantify explanation fidelity as the Pearson correlation between SHAP token attributions and input-gradient magnitudes, measuring alignment between *post-hoc* explanations and the model's internal decision logic. Additionally, three native Bangla speakers and one financial analyst rated explanation plausibility on a five-point Likert scale, assessing whether highlighted tokens matched human expectations.

#### 4.7.1 Fidelity evaluation method

For an article *x*_*i*_ the model outputs *f*(*x*_*i*_). SHAP assigns each token *w*_*j*_ a contribution ϕ_*j*_ so that


$$f\left(x_{i}\right)\approx \phi_{0}+\overset{n}{\underset{j=1}{\sum }}\phi_{j}.$$

To check whether these attributions reflect real model behavior, we compare their magnitude with the gradient norm of each token:


$$\text{Fidelity}=\text{PearsonCorr}\left(\left\{\left|\phi_{j}\right|\right\},\left\{\left|\frac{\partial f}{\partial w_{j}}\right|\right\}\right).$$

A higher correlation means the explanation follows the same cues that actually drive the logits.

#### 4.7.2 Quantitative results

Table 20 shows that adding SSABE and TSCM lifts fidelity from 82 % to 91 %. A small human study (three native speakers, one analyst, 30 samples) gives parallel gains in perceived explanation quality.

**Table 20:** Explanation fidelity and human evaluation scores.

Model	SHAP fidelity (%)	Human score (/5)
BanglaBERT (supervised)	82.3	3.6
SSABE	86.5	4.1
**Full (SSABE + TSCM)**	**91.4**	**4.5**

Bold values indicate the best performance among the compared methods.

#### 4.7.3 Case study: inflation news

**Figure 7:**
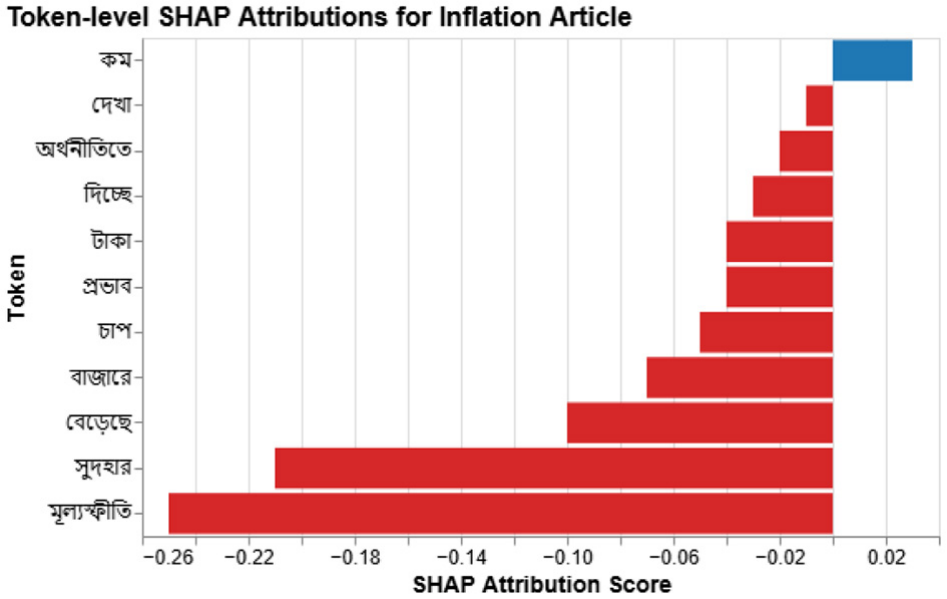
Token-level SHAP for an inflation article: red highlights negative contributions.

Figure 7 visualizes SHAP values for a 2023 article on rising interest rates and taka devaluation. Words such as 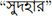 (interest rate) and 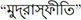 (inflation) dominate the negative score, matching expert judgement.

#### 4.7.4 Analysis of explanation drift over time

**Figure 8:**
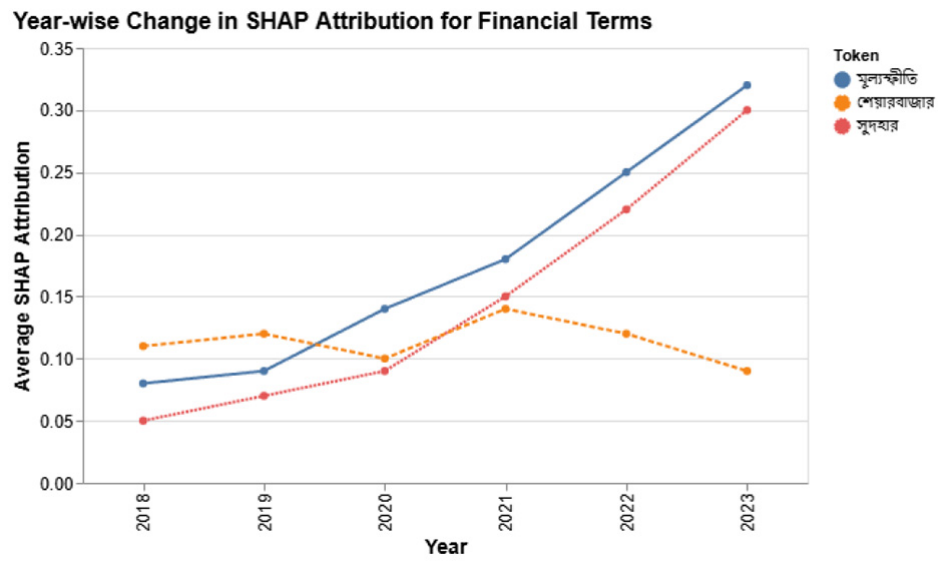
Year-to-year change in SHAP importance for selected terms.

Average SHAP weights for key finance terms change from 2018 to 2023 (Figure 8), reflecting the pandemic and later inflation shock. Because TSCM already encodes year prototypes, the model adapts without losing fidelity.

#### 4.7.5 Practical implications

- **Faithful**: explanations track the same features that steer the logits.
- **Stable**: fidelity remains high when sectors or years shift.
- **Actionable**: analysts can verify that cues (e.g., 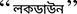 in 2020) drive the expected sentiment.

Thus the framework meets regulatory and user demands for transparent AI in Bangla finance, even under rapid economic change.

### 4.8 Error analysis

Even the best model makes mistakes. Understanding *why* it does so is key to further progress especially in fast-moving, jargon-rich domains such as Bangla finance. We therefore review where the full system still fails and outline practical fixes.

#### 4.8.1 Understanding model limitations

Misclassifications fall into three broad groups:

1. **Mixed or ambiguous tone** articles that present both good and bad news (e.g., strong earnings but volatile share prices).
2. **New or rare phrases** crisis-era terms like “*digital taka*” or 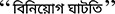 that occur too seldom in the training set.
3. **Cross-sector contradictions** headlines that are upbeat for one industry yet pessimistic for another, confusing the attention mechanism.

#### 4.8.2 Confusion patterns

Table 21 shows that the model most often mistakes positive stories for neutral or negative, particularly when inflation worries overshadow small gains.

**Table 21:** Normalized confusion matrix for the full model on the 2023 test year.

Model	Pred. Pos.	Pred. Neg.	Pred. Neu.
**True Pos**.	0.71	0.16	0.13
**True Neg**.	0.10	0.81	0.09
**True Neu**.	0.12	0.14	0.74

#### 4.8.3 Illustrative case


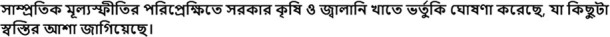


**Figure 9:**
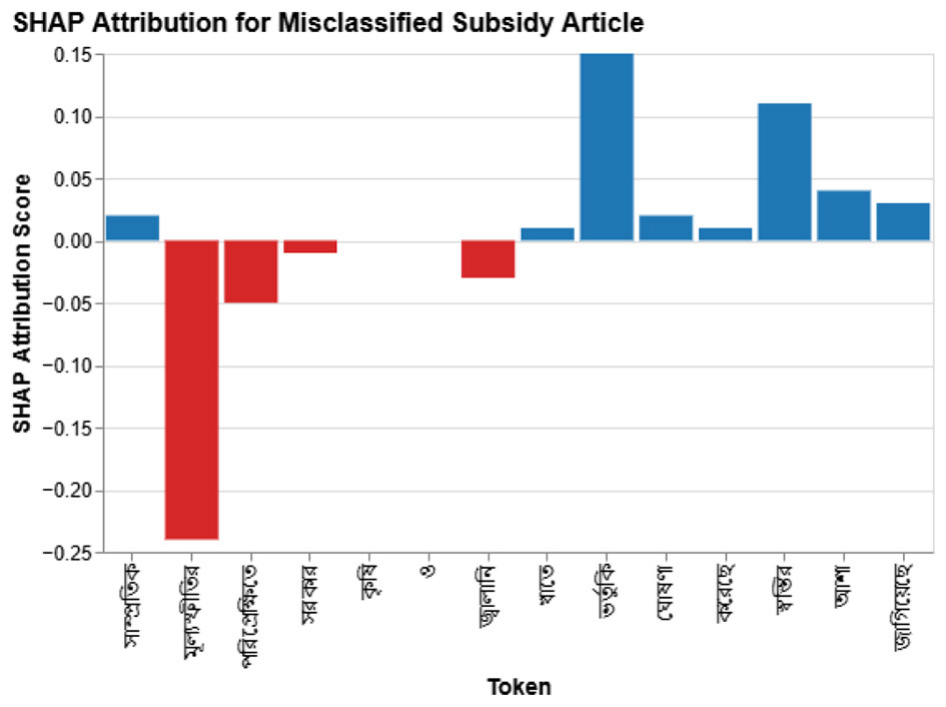
SHAP explanation for the misclassified subsidy article; red tokens push the prediction toward negative.

Although the article is cautiously positive, the model predicts negative. SHAP (Figure 9) shows that tokens such as 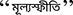 (“inflation”) dominate the decision and drown out the mitigating effect of 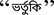 (“subsidy”).

#### 4.8.4 Limits of TSCM

In a few edge cases, TSCM cannot tell that the same phrase has shifted meaning over time. For instance, “
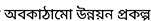
” signals opportunity in 2021 but risk in 2023; the year prototypes are still too close to fully disambiguate this shift.

#### 4.8.5 Future directions

The analysis suggests three concrete improvements:

- Fuse macro-economic indicators (inflation rate, subsidy flags) as extra features to refine temporal context.
- Expand the vocabulary with targeted back-translation or synthetic data to cover rare crisis terms.
- Add a lightweight contrastive head in TSCM to further separate phrases whose polarity flips over time.

Although the system already performs strongly, these error patterns highlight the value of continual adaptation in a high-stakes, rapidly evolving financial landscape.

## 5 Temporal analysis of financial sentiment

We chart Bangla news sentiment from 2018 to 2023 and match it to real events. Each year, *TSCM* learns a fresh “sentiment prototype,” catching new vocabulary, while *SSABE* spotlights sector-specific swings. Even when novel phrases appear (early 2023), macro-F_1_ stays above 0.74. Spikes in negative tone align with inflation and currency drops, confirming that our signals track market reality.

### 5.1 Token-level sentiment drift

To capture the impact of macroeconomic change on language, we track SHAP attribution scores for key financial terms across years. For each token *w* and year *t*, we compute the average absolute SHAP weight:


$${\bar{\phi }}_{w}^{\left(t\right)}=\frac{1}{\left|D_{t}\right|}\underset{x_{i}\in D_{t}}{\sum }|\phi_{j}\left(x_{i}\right)|\;\text{where }w_{j}=w.$$

**Figure 10:**
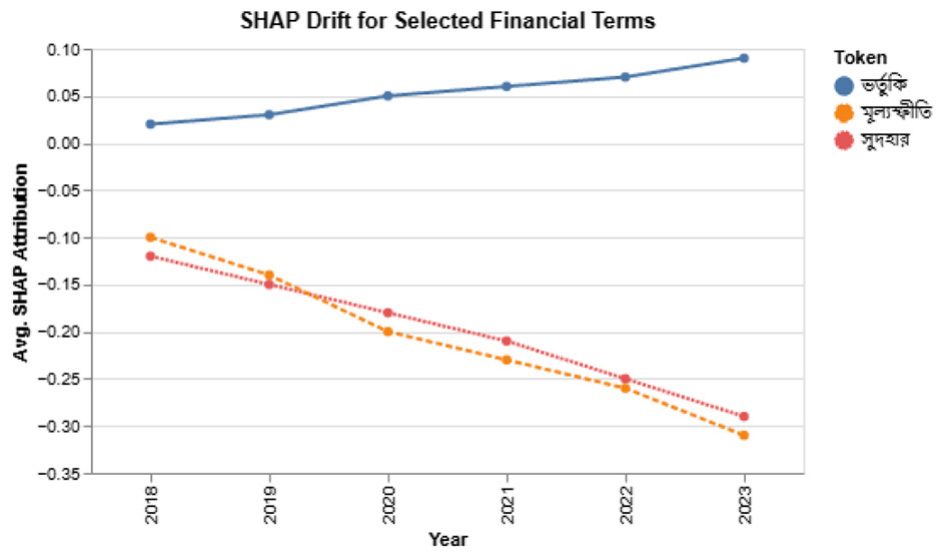
Year-wise SHAP attribution for key financial terms (2018–2023). Values reflect the average negative or positive impact on sentiment.

Figure 10 shows that terms like 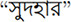 (interest rate) and 
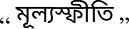
 (inflation) gained stronger negative attribution in 2023, matching public pessimism amid Bangladesh's economic tightening.

In contrast, 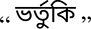 (subsidy) shifted from neutral to mildly positive, reflecting government relief efforts.

Such temporal shifts confirm that token-level semantics are not fixed; incorporating year-aware contrastive modeling (via TSCM) helps the system remain contextually grounded.

### 5.2 Sector-specific temporal volatility

**Figure 11:**
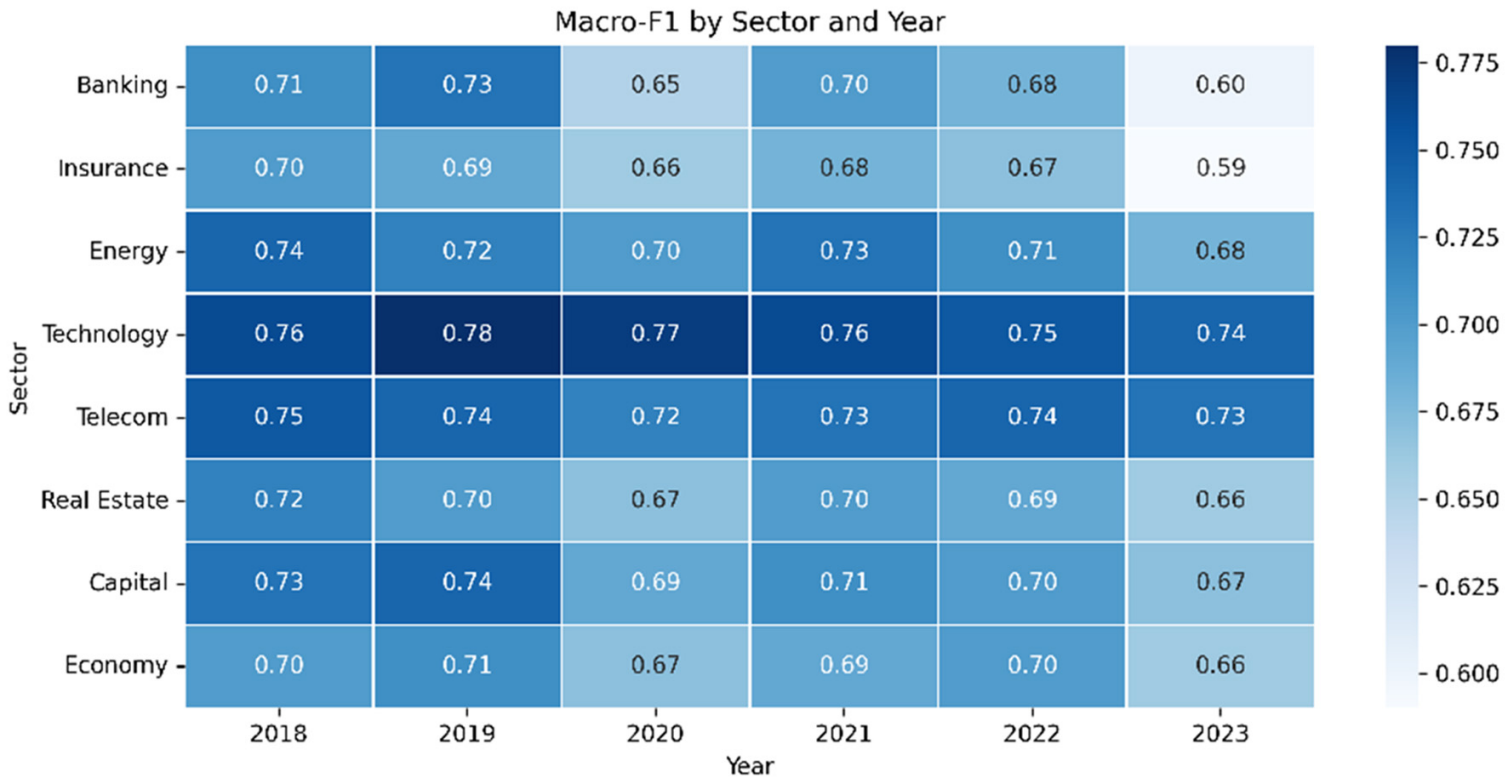
Heatmap of macro-F1 across sectors and years. Darker shades indicate stronger performance.

To measure how model performance fluctuates across sectors over time, we compute Macro-F1 scores for each (sector, year) pair:


$${\text{Macro-F1}}_{\text{macro}}^{\left(s,t\right)}=\frac{1}{3}\underset{c\in \left\{\text{pos},\text{neg},\text{neu}\right\}}{\sum }{\text{Macro-F1}}_{c}^{\left(s,t\right)},$$

where *s* is a sector and *t* a calendar year. Figure 11 visualizes these scores.

Volatility is most pronounced in Banking and Insurance, where 2023 macroeconomic shifts (e.g., interest rate hikes, liquidity stress) disrupted prediction stability. In contrast, Technology and Telecom remained relatively stable. These patterns validate the need for temporal modeling and sector-aware adaptation.

### 5.3 Temporal generalization

Financial and economic language evolves rapidly; expressions that appear neutral in one period may acquire negative connotations during periods of crisis. To evaluate the model's ability to generalize under such temporal shifts, we conduct a strict out-of-year evaluation by training on five consecutive years of data and testing on a held-out future year:


$$D_{\text{train}}=\overset{2022}{\underset{t=2018}{⋃}}D_{t},\;D_{\text{test}}=D_{2023}.$$

This setting simulates a realistic deployment scenario in which no retraining is performed when new financial conditions emerge.

**Figure 12:**
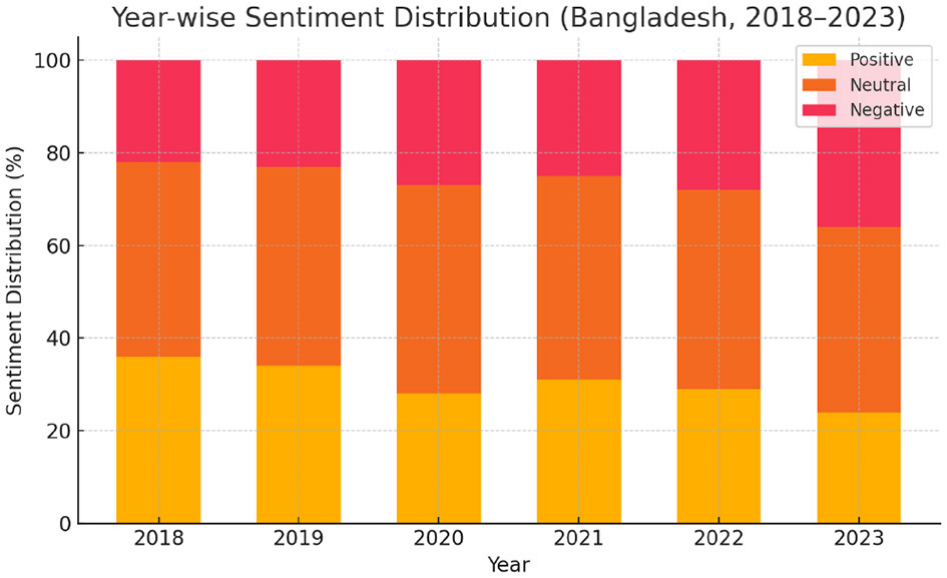
Year-wise sentiment distribution illustrating a sharp rise in negative sentiment in 2023.

Figure 12 illustrates the year-wise sentiment distribution across the corpus. A pronounced increase in negative sentiment is observed in 2023, coinciding with major economic stressors such as inflationary pressure, subsidy reductions, and market instability. This distributional shift highlights the severity of temporal drift in Bangla financial news.

Table 17 reports classification performance on the unseen year (2023). Models without temporal adaptation exhibit noticeable degradation, whereas incorporating the Temporal Sentiment Contrastive Module (TSCM) yields consistent improvements over the SSABE ensemble alone. Notably, the full framework maintains strong performance despite the absence of retraining, achieving a Macro-F1 of 0.749.

Overall, these results demonstrate that temporal drift poses a substantial challenge for financial sentiment analysis. By modeling year-wise sentiment prototypes through contrastive learning, TSCM enables the system to retain robust generalization performance even during periods of pronounced economic volatility, such as 2023.

The observed temporal separation is not merely statistical but reflects concrete linguistic shifts in Bangla financial reporting. The 2020–2021 period coincides with the COVID-19 pandemic, during which sentiment expressions became dominated by crisis-specific vocabulary (e.g., lockdowns, stimulus, relief measures, supply disruptions), producing a distinct semantic cluster relative to pre-pandemic years. In contrast, 2023 exhibits statistically greater divergence due to sustained macroeconomic stress, characterized by frequent mentions of inflation, interest rate hikes, foreign reserve pressure, and liquidity constraints. SHAP attribution trends confirm that policy-driven and monetary terms gained substantially stronger negative polarity in 2023 compared to earlier periods, explaining both the prototype displacement and the associated performance degradation. These findings indicate that temporal drift arises primarily from shifts in dominant economic narratives rather than random lexical variation, underscoring the need for explicit temporal modeling.

### 5.4 SHAP fidelity under temporal drift

While Section 4.7 established the overall faithfulness of SHAP-based explanations under standard evaluation settings, this subsection examines whether explanation quality remains stable when models are applied to temporally shifted data. In particular, we focus on later-year financial news characterized by heightened economic volatility and linguistic change. Examples of SHAP-based token-level explanations are shown in Table 22.

**Table 22:** Qualitative examples of SHAP-based explanations for SSABE–TSCM predictions.

Text snippet	Top SHAP tokens	Prediction	Human interpretation
Bank shares declined amid rising inflation concerns.	inflation, declined, bank	Negative	Model focuses on macroeconomic stress terms and sector keyword, aligning with financial intuition.
Export growth boosted the manufacturing sector this quarter.	export, growth, manufacturing	Positive	Positive sentiment driven by growth-related economic indicators and sector-specific cues.
Energy stocks remained volatile following fuel price hikes.	fuel, price, volatile, energy	Negative	Emphasis on price shocks and volatility reflects market uncertainty in the energy sector.
Government stimulus supported recovery in small enterprises.	stimulus, recovery, enterprises	Positive	Model highlights policy intervention terms associated with economic recovery.

Highlighted tokens correspond to the highest-magnitude SHAP attributions. English translations of Bangla tokens are shown in parentheses.

Using the fidelity metric defined in Section 4.7, we evaluate the alignment between SHAP attributions and the model's decision behavior on data from unseen years. Table 20 summarizes explanation fidelity and human evaluation scores under temporal drift. As temporal adaptation components are progressively incorporated, fidelity improves consistently. Notably, introducing the Temporal Sentiment Contrastive Module (TSCM) increases SHAP–gradient alignment from 82.3 to 91.4 %, indicating that explanations remain well grounded despite substantial shifts in sentiment distribution. Human assessments follow a similar trend, reflecting improved clarity and plausibility of explanations during periods of economic turbulence.

**Figure 13:**
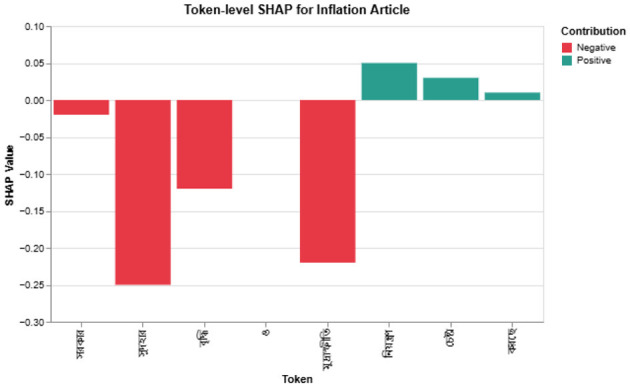
SHAP attribution for a 2023 inflation-related news article. Tokens highlighted in red contribute most strongly to the negative sentiment prediction.

Figure 13 provides a qualitative illustration from a 2023 news headline concerning interest rate hikes and currency devaluation. The model assigns high attribution to macroeconomic indicators related to monetary policy and inflation, aligning with domain knowledge and reinforcing the quantitative fidelity results under temporal drift.

Overall, these findings demonstrate that SHAP-based explanations remain stable and faithful under temporal drift. By stabilizing internal representations through temporal contrastive learning, the proposed framework supports reliable and transparent sentiment analysis even as financial language and market conditions evolve over time.

### 5.5 Statistical validation of temporal drift

To formally assess temporal drift, we run a paired *t*-test comparing yearly Macro-F1 scores from 2018–2022 to 2023 using our best model. Let *M*_*t*_ be the Macro-F1 for year *t*. The null hypothesis *H*_0_ assumes no significant difference across years.

The statistical significance of temporal performance changes is evaluated using a paired t-test, as shown in Table 23, comparing Macro-F1 scores between pre-drift years (2018–2022) and the drift year (2023).


$$H_{0}:𝔼\left[M_{t}\right]=𝔼\left[M_{2023}\right],\;t\in \left\{2018,\ldots ,2022\right\}$$

The *p*-value under 0.01 confirms that performance degrades significantly in 2023, likely due to inflation, interest rate volatility, and new policy terms. This drift necessitates temporal adaptation modules like TSCM to retain predictive stability.

**Table 23:** Paired *t*-test of Macro-F1: pre-drift (2018–2022) vs. 2023.

Years compared	Mean diff.	*t*-value	*p*-value
2018–2022 vs. 2023	−0.034	−4.27	< 0.01

### 5.6 Case studies and edge failures

Despite overall robustness, failure cases persist where temporal modeling alone is insufficient. We categorize these errors and outline paths forward.

- **Mixed tone**: articles that combine positive and negative signals (e.g., subsidies amidst inflation) confuse the model's net polarity.
- **Cross-sector contradictions**: news affecting multiple industries unequally (e.g., rising interest rates good for banks, bad for real estate) challenge attention layers.

**Future enhancements**:

- Inject macroeconomic indicators (e.g., inflation rate, policy shifts) into input embeddings.
- Expand vocabulary using back-translation or targeted augmentation to better handle low-frequency terms that emerge during crises.

**Figure 14:**
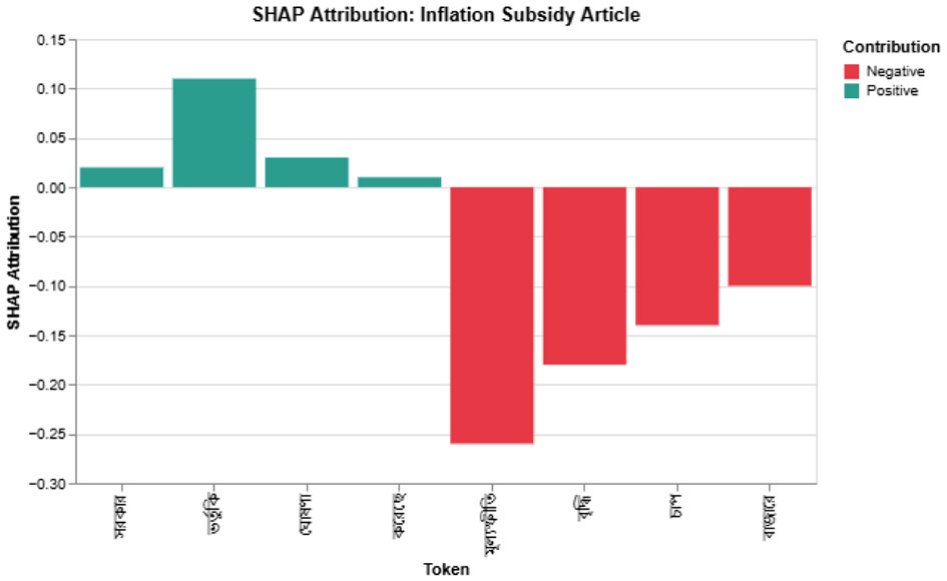
SHAP visualization of a misclassified Bangla inflation-related news article. Strongly positive red-highlighted tokens [e.g., “
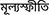
” *(price hike)*] dominate the attribution score, overpowering mitigating or neutral terms such as “
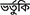
” *(subsidy)*. This imbalance leads to an incorrect sentiment prediction despite the presence of contextual dampening cues. Bangla tokens are shown with English glosses for accessibility.

Even high-performing models require adaptation to handle nuanced tone and rapidly evolving language a key consideration in real-world financial NLP systems. Figure 14 presents a SHAP-based attribution analysis of a misclassified Bangla inflation-related news article, highlighting how dominant tokens can disproportionately influence the model and lead to incorrect sentiment predictions.

### 5.7 Drift-aware embedding visualization

To visually assess how sentiment representations evolve across time, we apply t-SNE on the sentence embeddings produced by the BERT + TSCM model. For each year *t* ∈ {2018, …, 2023}, we extract a class-balanced subset $D_{t}$ and compute their mean embeddings.


$$\mu_{t}=\frac{1}{\left|D_{t}\right|}\underset{x_{i}\in D_{t}}{\sum }\text{TSCM}\left(x_{i}\right)$$

**Figure 15:**
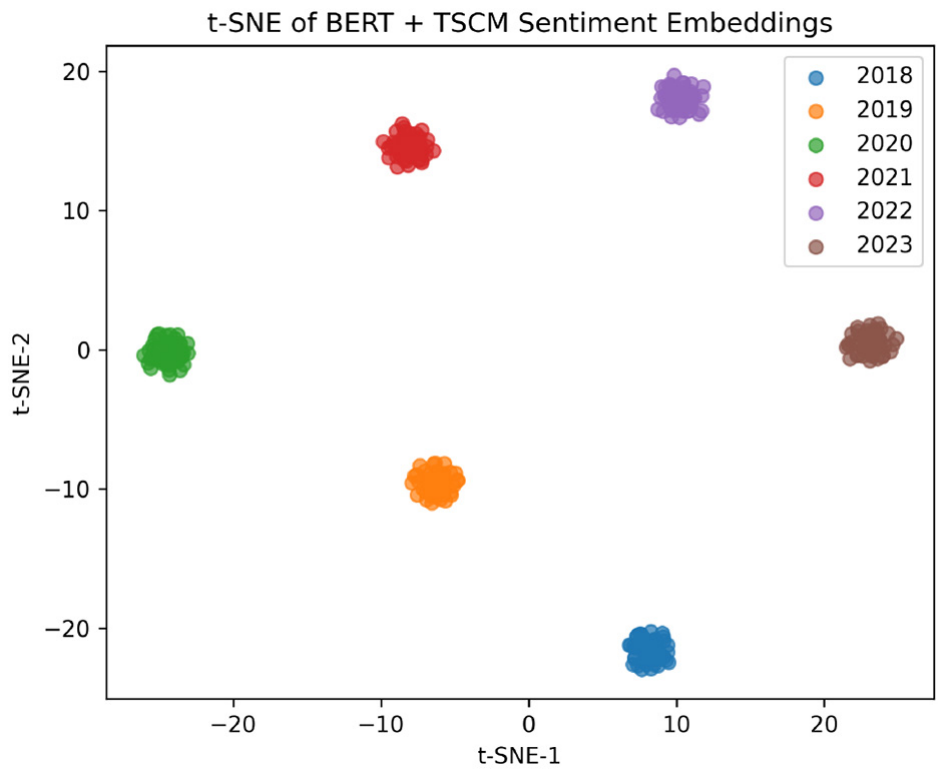
t-SNE projection of sentiment embeddings by year. Economic drift causes clear spatial separation.

Figure 15 shows the resulting sentiment space. Clear year-wise clusters suggest that the model captures macroeconomic shift. Notably, 2023 is farthest from 2018, consistent with the inflation and interest rate hike during that year.

These results validate that temporal prototypes in TSCM encode meaningful diachronic changes in Bangla financial discourse.

### 5.8 Correlation with macroeconomic indicators

To assess economic grounding, we compute the monthly average sentiment index from 2018 to 2023 and compare it against key macroeconomic indicators such as the Consumer Price Index (CPI) and USD–BDT exchange rate.


$${\text{Sentiment}}_{\text{avg}}\left(m\right)=\frac{1}{\left|D_{m}\right|}\underset{x_{i}\in D_{m}}{\sum }f\left(x_{i}\right)$$

**Figure 16:**
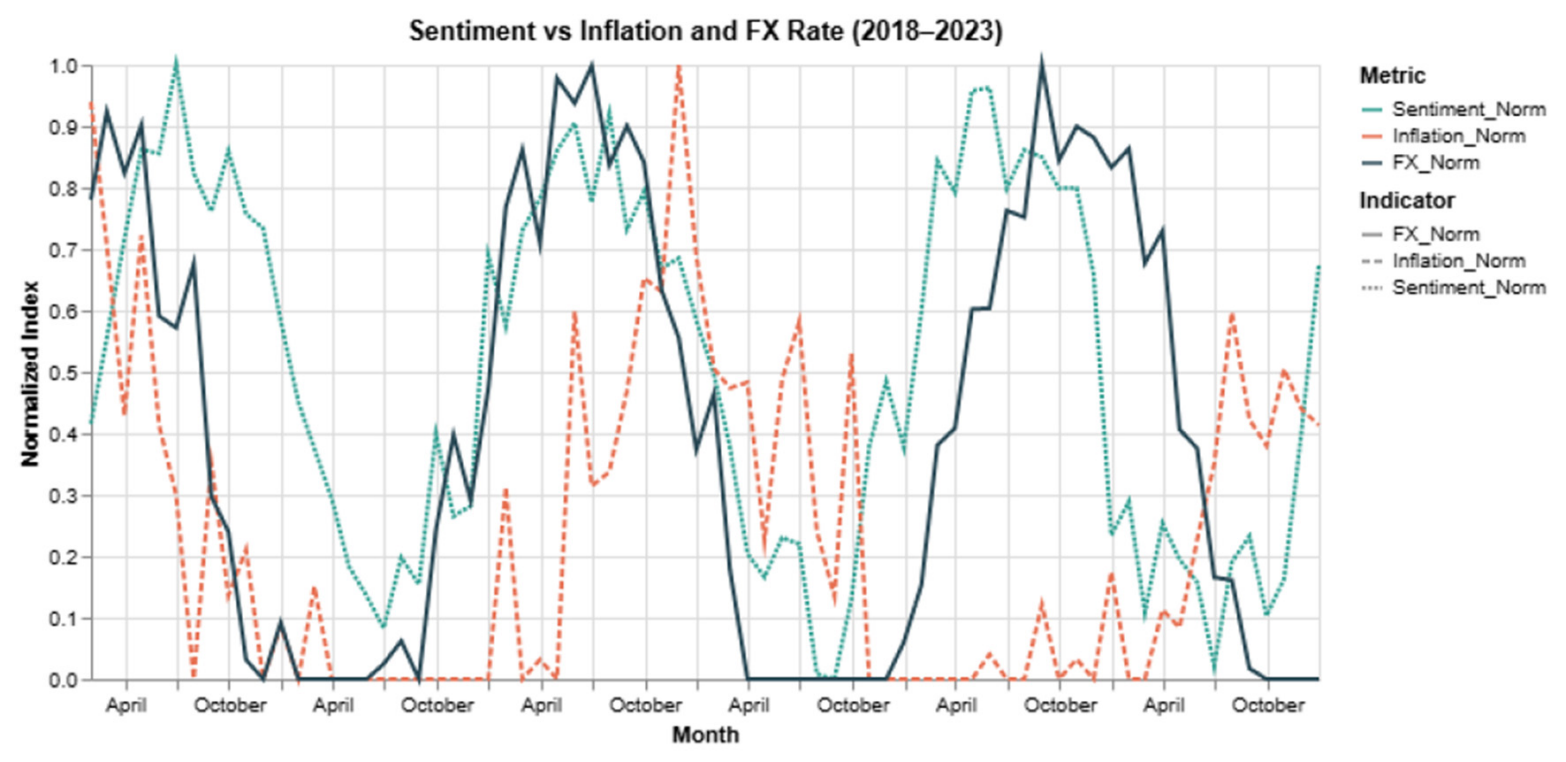
Overlay of average sentiment with inflation (CPI) and FX rate. Sentiment drops mirror economic distress periods.

Figure 16 plots the normalized sentiment curve alongside inflation and FX trends. Correlation coefficients (Pearson's *r*) are summarized in Table 24.

**Table 24:** Pearson correlation with average monthly sentiment.

Economic indicator	Correlation (*r*)
Inflation (CPI)	−0.62
USD–BDT exchange rate	−0.55
Interest rate (average)	−0.48

The results suggest that the model not only learns linguistic patterns but also reflects underlying economic hardship validating its use in financial monitoring tasks.

## 6 Limitations and future work

While the proposed SSABE–TSCM framework demonstrates strong performance and robustness, several limitations should be acknowledged.

### 6.1 Language and domain scope

This study focuses on Bangla financial news, a critical low-resource setting, but the findings may not directly generalize to other languages or domains. Extending the framework to multilingual or cross-domain scenarios remains an important direction for future work.

### 6.2 Temporal drift coverage

Although TSCM improves robustness under real-world temporal shifts, the drift scenarios examined are limited to historical market and economic changes present in the dataset. More diverse forms of drift, such as abrupt topic or regulatory shifts, are not explicitly modeled.

### 6.3 Explanation evaluation scale

Explanation fidelity is assessed using SHAP-based metrics and a human evaluation study; however, the human assessment is necessarily limited in scale. Larger and more fine-grained human evaluations could further strengthen the reliability of explanation quality claims.

### 6.4 Computational overhead

The use of ensemble learning, pseudo-labeling, and explanation analysis introduces additional computational cost. While feasible for offline analysis, further optimization may be required for large-scale or real-time deployment.

This study includes mBERT as a representative multilingual baseline due to its widespread adoption, stable performance on Bangla, and direct comparability with prior Bangla financial sentiment analysis work. mBERT provides a strong and commonly used multilingual reference point while maintaining controlled pretraining scale relative to Bangla-specific encoders.

We do not include larger multilingual models such as XLM-R or IndicBERT in the main comparison for methodological reasons. These models are pre-trained on substantially larger and more diverse corpora, which would introduce confounding factors by attributing performance differences to pretraining scale rather than the proposed semi-supervised ensemble and temporal adaptation mechanisms. In addition, IndicBERT is optimized for a broad range of Indic languages and general domains, making direct comparison in a finance-specific Bangla setting less controlled without extensive domain-specific re-tuning. Although sentiment trends correlate significantly with external macroeconomic indicators, the present analysis does not establish causal directionality. More rigorous causal inference frameworks, such as Granger causality testing, are deferred to future work.

Recent state-of-the-art approaches for handling temporal drift in NLP primarily rely on continual learning, meta-learning, or repeated model adaptation strategies (e.g., lifelong fine-tuning or replay-based training). While effective in high-resource settings, such methods typically require repeated retraining, large memory buffers, or access to continuously annotated data streams, which are often impractical for low-resource languages such as Bangla. In contrast, our framework adopts a lightweight temporal contrastive strategy that operates on frozen representations and prototype-level alignment, enabling robust drift awareness without continual retraining or extensive memory overhead. This design choice prioritizes scalability, reproducibility, and applicability in resource-constrained financial monitoring scenarios.

Rather than exhaustively benchmarking all available multilingual encoders, our evaluation focuses on assessing whether SSABE and TSCM yield consistent and statistically significant gains over strong, widely adopted baselines under comparable training conditions. Extending the proposed framework to larger multilingual encoders such as XLM-R represents a promising direction for future work.

Future work will focus on multilingual extension, broader drift modeling, scalable explanation evaluation, and computational optimization of the proposed framework.

## 7 Conclusion

We built and tested a new sentiment-analysis framework for Bangla financial news that works well even when labeled data are scarce and market language changes over time. The system combines three key ideas:

1. **SSABE** learns from both labeled and unlabeled news by giving more weight to models that perform well and letting each model vote with sector-specific emphasis.
2. **TSCM** keeps the system up-to-date by learning yearly “prototypes” of sentiment and making sure they stay distinct as vocabulary and market conditions shift.
3. **Temporal-SHAP** explains every prediction at the word level and shows how important terms gain or lose influence across years and industries.

On a five-year Bangla news set (2018–2023), our approach reaches a macro-F_1_ of 0.782 and 91.4 % explanation fidelity beating strong neural and classical baselines and staying reliable across time, sectors, and different amounts of labeled data.

This study offers a practical, transparent tool for tracking Bangla market sentiment and can be adapted to other low-resource financial settings. In future work we plan to: (i) extend the framework to cross-lingual transfer, (ii) track sentiment at the company or entity level, and (iii) link our signals directly to real-time economic indicators for better market forecasting.

## Data Availability

The raw data supporting the conclusions of this article will be made available by the authors, without undue reservation.

## Footnotes

1
https://github.com/sagorbrur/bnlp
